# Effect of Titanium Dioxide (TiO_2_) Incorporation on the Properties of Glass Ionomer Cements: A Systematic Review

**DOI:** 10.3390/ma19132827

**Published:** 2026-07-02

**Authors:** Julia Kensy, Agnieszka Kotela, Jakub Wenderski, Agata Małyszek, Maciej Dobrzyński, Jacek Matys

**Affiliations:** 1 Department of Pediatric Dentistry and Preclinical Dentistry, Wroclaw Medical University, Krakowska 26, 50-425 Wroclaw, Poland; maciej.dobrzynski@umw.edu.pl; 2 Medical Center of Innovation, Wroclaw Medical University, Krakowska 26, 50-425 Wroclaw, Poland; kotela.agnieszka@gmail.com; 3 4th Military Teaching Hospital, Weigla 5, 50-981 Wroclaw, Poland; k.wenderski@gmail.com; 4 Department of Biostructure and Animal Physiology, Wrocław University of Environmental and Life Sciences, Kozuchowska 1, 51-631 Wroclaw, Poland; agata.malyszek@upwr.edu.pl; 5 Dental Surgery Department, Wroclaw Medical University, Krakowska 26, 50-425 Wroclaw, Poland

**Keywords:** glass ionomer, titanium dioxide, TiO_2_, titanium oxide, restoration material

## Abstract

This systematic review aimed to investigate the effect of titanium dioxide (TiO_2_) incorporation on the mechanical, physicochemical, and biological properties of conventional glass ionomer cements (GICs). A systematic search was conducted in June 2026 in PubMed, Scopus, Embase, Web of Science and WorldCat databases. Search terms included combinations of glass ionomer AND titanium dioxide OR TiO_2_ OR titanium oxide OR titanium nanotubes OR titanium nanoparticles. The study selection process followed the PRISMA guideline and was organized according to the PECO framework. The search yielded the identification of 475 articles, of which 34 met the eligibility criteria. The included studies investigated different TiO_2_ forms, concentrations, and commercial GIC formulations. Many studies reported improvements in compressive strength, surface microhardness, fracture toughness, and antibacterial activity following TiO_2_ incorporation. However, the findings were heterogeneous. Several studies reported no statistically significant differences or contradictory outcomes, particularly regarding flexural strength, fluoride release, cytocompatibility, and antibacterial performance. Beneficial effects were most frequently observed at TiO_2_ concentrations between 3 and 5 wt%, whereas higher concentrations were occasionally associated with nanoparticle agglomeration and reduced material performance. Variability among studies was likely influenced by differences in TiO_2_ characteristics, concentration, testing protocols, and GIC formulation. Overall, TiO_2_ incorporation appears to be a promising approach for enhancing selected properties of conventional GICs. However, further standardized studies are required to confirm the consistency and clinical relevance of these effects.

## 1. Introduction

Glass ionomer cement (GIC) is a self-adhesive restorative material. It is composed of a fluoroaluminosilicate glass powder and an aqueous solution of polyacrylic acid. These components react through an acid–base setting reaction forming a hardened cement matrix [1,2]. This material is one of the most popular used in dentistry especially as restorative material, luting material or sealing material [3,4,5]. It exhibits excellent properties which have led to its widespread use in many fields of dentistry. One of the characteristic features of glass ionomer cements is their ability to chemically bond to dentin, which leads to durable adhesion to tooth structures, enhances marginal sealing, reduces microleakage and postoperative sensitivity [6,7,8]. Furthermore, they possess fluoride-release and fluoride-recharge capabilities, which gives them additional anti-caries properties [9,10,11]. GICs also exhibit excellent biocompatibility and favorable pulp responses, making them particularly suitable for use in pediatric dentistry and in patients at high risk of caries [12]. However, despite this numerous advantages, conventional glass ionomer cements have several important limitations [13,14]. Their low fracture toughness and poor flexural strength, reduce their ability to withstand functional stress [15,16]. Secondly, conventional glass ionomer cements are particularly vulnerable to moisture contamination during the early stages of setting, which can interfere with the acid–base reaction and consequently impair their mechanical properties and long-term durability [17]. Additionally, their use does not provide a satisfactory esthetic result, due to limited translucency and polishability [18]. These limitations have prompted research aimed at developing innovative strategies to strengthen and improve glass ionomer materials and enhance their properties, thereby improving their clinical performance.

To overcome the drawbacks of conventional glass ionomer cements and achieve better clinical outcomes, extensive research has been conducted to investigate possible modifications that could improve their properties [19,20,21,22]. One of the most important steps was the development of resin-reinforced glass ionomer cements (RMGIC) [23,24]. The incorporation of resin monomers, particularly 2-hydroxyethyl methacrylate (HEMA), enabled these materials to undergo both the traditional acid–base setting reaction and a light-activated polymerization reaction, resulting in improved mechanical strength, greater wear resistance, reduced moisture sensitivity, and enhanced esthetic properties [25,26,27]. Recent advances in nanotechnology have also enabled the incorporation of bioactive ceramics, fibers, and inorganic fillers to create hybrid structures with increased strength [28,29,30]. The incorporated nanoparticles better fill the spaces between cement particles more effectively, increasing the density of the cement matrix and improving stress distribution throughout the material, potentially resolving the trade-off between mechanical strength and the biological benefits of traditional GIC formulations [31,32,33,34]. Another promising strategy is the incorporation of metallic additives such as silver, strontium, zinc, and various metal oxides into the glass ionomer cement matrix [10,35,36]. These elements can improve the mechanical properties, by increasing compressive strength or flexural resistance [13,37]. In addition, the incorporation of certain metallic ions, especially strontium and zinc increase radiopacity of the cement, facilitating radiographic assessment [38,39]. Many of metallic additives also exhibit antibacterial and bioactive properties, which may inhibit the growth of cariogenic microorganisms, and contribute to improved long-term clinical outcomes [10,40,41].

**Figure 1:**
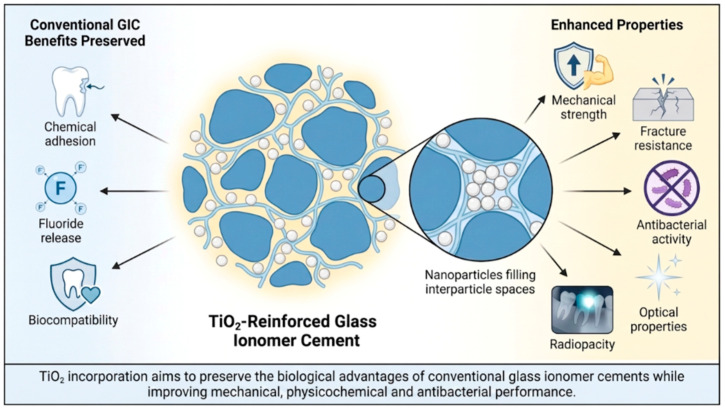
Conceptual illustration of the potential effects of TiO_2_ incorporation into glass ionomer cements. (Created in BioRender. Kensy, J. (2026) https://BioRender.com/xxhdz72).

In terms of metallic additives, titanium dioxide (TiO_2_) has emerged as a promising reinforcing agent for conventional glass ionomer cements [42,43,44]. Owing to its favorable chemical stability and good biocompatibility performance, TiO_2_ is considered highly resistant to the challenging conditions of the oral environment [45,46,47]. Advances in nanotechnology have further increased the interest in this material, as various forms of titanium dioxide, such as nanoparticles and nanotubes, can provide a significantly higher surface-to-volume ratio compared with conventional particles [48,49]. This enhanced area promotes stronger interaction between TiO_2_ particles and the cement matrix, which in turn may improve fracture resistance and reduce material fragility [50]. Beyond its mechanical benefits, titanium dioxide also exhibits notable antibacterial activity, contributing to the inhibition of microbial growth [51,52]. Additionally, it exhibits satisfactory optical properties such as high refractive index and natural white hue, which may enhance the esthetic performance of glass ionomer restorations, aligning with the demands of modern restorative dentistry [53,54]. Overall, the incorporation of TiO_2_ into glass ionomer cements aims to develop a hybrid material that preserves the intrinsic advantages of fluoride release while offering improved mechanical strength, particularly for use in stress-bearing posterior restorations (see Figure 1) [55].

Despite the large number of studies evaluating different modifications of glass ionomer cements, a comprehensive synthesis of the TiO_2_ incorporation effect is still lacking in the present literature. Many individual reports have already described the advantages of this modification, and improvements in mechanical, physicochemical, antibacterial and biological properties. However, the findings are not homogeneous and are strongly influenced by factors related to TiO_2_ itself such as particle size and morphology, concentration and type of used GIC. Therefore, there is a need to systematically evaluate the available data to understand the real impact on glass ionomer cements reinforcement. This systematic review aims to address this gap by critically analyzing and integrating the current literature on TiO_2_-modified GICs.

## 2. Materials and Methods

### 2.1. Focused Question

The systematic review was conducted in accordance with the PECO framework [56]. In glass ionomer cements used in dental applications (P), does the incorporation of titanium dioxide (E), compared with conventional unmodified formulations (C), lead to measurable improvements in their mechanical, physical, antibacterial, and biological properties (O)?

### 2.2. Protocol

**Figure 2:**
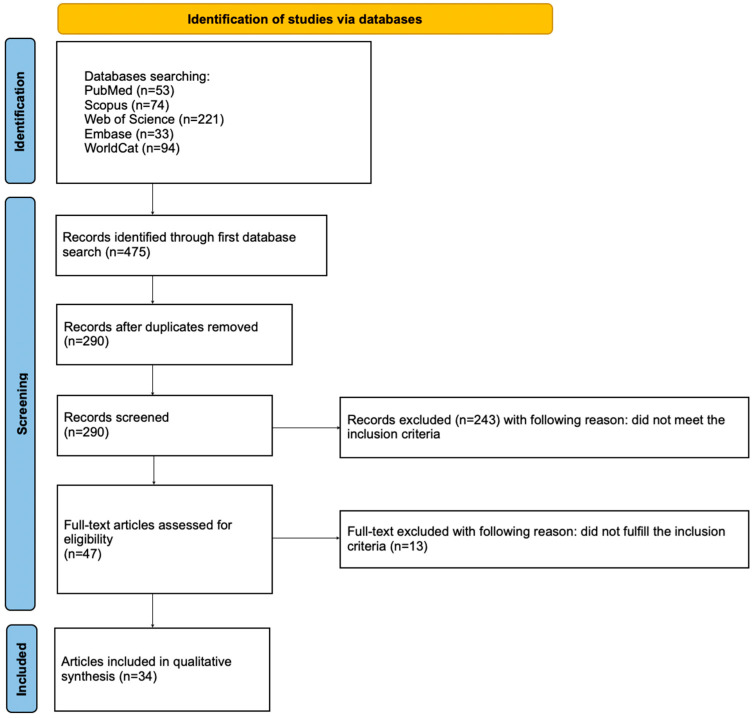
The PRISMA 2020 flow diagram [57].

The process of identifying and selecting studies adhered to the PRISMA methodology, as depicted in Figure 2 [57]. The review protocol was prospectively registered on the Open Science Framework (OSF) under the following link: https://osf.io/bhj5x/overview (assessed on 15 May 2026) (Supplementary Table S1).

### 2.3. Eligibility Criteria

Studies were included in this review according to the following criteria [58,59]:

- In vitro experimental studies evaluating glass ionomer cements modified with titanium dioxide (TiO_2_)
- Studies evaluating modifications of conventional glass ionomer cements (defined as acid–base setting materials without resin components)
- Studies including a control group
- Studies assessing at least one mechanical, physical, chemical, or antimicrobial property of the material
- Studies published in English

The restriction to English-language publications was applied due to resource limitations and may have introduced language bias.

Exclusion criteria established by the reviewers included [58,59]:

- Studies evaluating cements other than conventional glass ionomer cements (including resin-modified glass ionomers (RMGIC))
- Studies that evaluated additives other than titanium dioxide
- Studies lacking a control group of unmodified glass ionomer cement
- Clinical studies, in vivo studies,
- Clinical case reports;
- Expert opinions or commentaries;
- Editorials;
- Review papers;
- Duplicate publications.

Full texts were obtained through institutional subscriptions, open-access sources, and other available academic resources. Studies for which the full text could not be retrieved after reasonable efforts were not included in the qualitative synthesis. No restrictions on the year of publication were applied.

### 2.4. Information Sources, Search Strategy, and Study Selection

A comprehensive electronic search was conducted in June 2026 across multiple databases, including PubMed, Scopus, Web of Science (WoS), Embase, and WorldCat. Additionally, hand-searching of the reference lists of included studies and relevant reviews was conducted. The aim was to identify all relevant in vitro studies investigating the effects of incorporating titanium dioxide nanoparticles into conventional glass ionomer cements. The database search involved using a combination of controlled vocabulary and free-text terms related to glass ionomers and titanium dioxide. The general Boolean query applied was: ((glass ionomer) AND (titanium dioxide OR TiO_2_ OR titanium oxide OR titanium nanotubes OR titanium nanoparticles)). Database-specific search strings were presented in Table 1.

### 2.5. Data Collection Process and Data Items

Three independent reviewers (J.K., A.K., and J.W.) conducted the screening and selection of studies in accordance with the predefined inclusion criteria. From each eligible study, key information was extracted, including the first author, year of publication, study design, article title, titanium oxide addition results, and glass ionomer used. For studies evaluating multiple material modifications, only data derived from experimental groups directly comparing conventional GICs with TiO_2_-modified GICs were extracted and included in the qualitative synthesis. Experimental groups containing additional modifiers were not considered when attributing effects specifically to TiO_2_ incorporation. All extracted data were systematically organized and recorded in a standardized Microsoft Excel 365 spreadsheet (Version 2505, Build 16.0.18827.20102, 64-bit).

### 2.6. Risk of Bias and Methodological Quality Assessment

During the preliminary screening stage, all assessors independently evaluated the titles and abstracts of the identified records to minimize the possibility of selection bias. Inter-reviewer agreement was measured using Cohen’s kappa coefficient, and any disagreements regarding study inclusion or exclusion were resolved through discussion until consensus was reached.

Two blinded reviewers (J.M. and M.D.) independently assessed the methodological quality of the included studies using the QUIN Tool [60]. This instrument includes 12 criteria addressing key methodological domains. Each item was scored as “adequately specified” (score = 2), “inadequately specified” (score = 1), or “not specified” (score = 0). For each study, the final QUIN score was calculated as follows:

Final score (%) = (Total score obtained × 100)/(2 × number of applicable criteria).

Based on the final percentage score, studies were categorized as having a low risk of bias (>70%), medium risk of bias (50–70%), or high risk of bias (<50%).

The previous statement that all included studies achieved a perfect quality score has been removed. In the revised version, the quality assessment shows that most included studies were classified as having a medium risk of bias, while only three studies were categorized as having a low risk of bias. Inter-reviewer agreement was assessed using Cohen’s kappa coefficient calculated with MedCalc software (version 23.1.7; MedCalc Software Ltd., Ostend, Belgium). The resulting kappa value was 0.84 (*p* < 0.001), indicating excellent agreement between reviewers.

## 3. Results

### 3.1. Study Selection

A systematic database search was conducted across PubMed, Scopus, Embase, Web of Science, and WorldCat, yielding a total of 475 records. Following the removal of duplicate entries, 290 articles remained and were screened based on their titles and abstracts. During this phase, 243 publications were excluded as they did not address TiO_2_-modified glass ionomer cements or did not report outcomes relevant to the scope of this review. The full texts of the remaining 47 articles were then retrieved and evaluated against the predefined eligibility criteria, resulting in the exclusion of a further 13 studies. Consequently, 34 studies met the eligibility criteria and were included in the qualitative synthesis [48,61,62,63,64,65,66,67,68,69,70,71,72,73,74,75,76,77,78,79,80,81,82,83,84,85,86,87,88,89,90,91,92,93].

Due to substantial methodological heterogeneity among the included studies, statistical pooling of the data was considered inappropriate. The studies differed considerably with respect to the type of TiO_2_ incorporated into the glass ionomer cement, including nanoparticles, nanotubes, microparticles, and biosynthesized particles; TiO_2_ concentration; type and commercial brand of glass ionomer cement; powder-to-liquid ratio; storage medium and storage duration; testing protocols and standards; and methods used to measure outcomes. Furthermore, considerable variation existed in the evaluated properties, including mechanical, physical, antibacterial, and biological characteristics. These differences limited the comparability of results across studies and precluded a meaningful meta-analysis.

### 3.2. General Characteristics of the Included Studies

All studies included in this review were conducted in vitro [48,61,62,63,64,65,66,67,68,69,70,71,72,73,74,75,76,77,78,79,80,81,82,83,84,85,86,87,88,89,90,91,92,93]. Most of the studies evaluated conventional glass ionomer cements, commercially available on the market. Mostly used GIC were Ketac Molar EasyMix [48,62,69,74,75,77,78,80,81,83,91] and Fuji IX or Fuji IX Extra [62,65,68,75,76,84] with several studies also using Fuji IX Gold Label [72], Fuji II [71,85,89,93], Fuji Triage [90], Ketac Universal [90] Equia Fil [64,87], ChemFil Rock [64,87], Riva self-cure [82], Ketac Cem Radiopaque [83], Ionofil U [92] or Kromoglass 2 [66]. Additionally, several studies evaluated GICs modified with experimentally biosynthesized TiO_2_ nanoparticles obtained from probiotic bacteria [72,73] or plant extracts [83,84,85].

The included studies tested a broad range of additive concentrations varying between 1 wt% and 10 wt% [48,61,62,63,64,65,67,68,69,70,71,72,73,74,75,86,87,88,89,90,91,92,93] with 5 wt% being the most frequently investigated concentration and generally yielding the most favorable outcomes. The heterogeneous aims of the studies reflected the multifaceted nature of GIC research. The largest subset focused on mechanical properties, evaluating parameters such as compressive strength, flexural strength, surface microhardness, fracture toughness, and wear resistance [48,61,63,64,65,67,68,69,72,73,76,79,82,83,84,85,86,87,88,90,92,93]. Several investigations focused on physicochemical and structural properties, such as water sorption and solubility, fluoride release and recharge capacity, ion release profiles, microstructural features analyzed by scanning electron microscopy and energy-dispersive X-ray spectroscopy [48,62,63,64,65,66,71,72,73,75,80,82,84,86,87,88,90,91,92]. A few studies assessed biological outcomes, including cytotoxicity, cell viability, antibacterial activity against cariogenic organisms (*Streptococcus mutans* and *Lactobacillus acidophilus*), and biofilm inhibition [48,62,65,67,70,71,74,75,77,78,79,81,82,85,86,88]. A smaller subset of studies addressed outcomes of clinical relevance, such as radiopacity, working time, adhesion to dentin and color stability [77,78,81,85,91]. Notably, studies by Meyer et al. and Rangel-Coelho et al. assessed the immunomodulatory and inflammatory marker expression of pre-odontoblastic cells in the presence of TiO_2_-modified GIC, representing a more advanced biological evaluation approach [74,77]. The general characteristic of the included studies is presented in Supplementary Table S2.

### 3.3. Main Study Outcomes

#### 3.3.1. Mechanical Properties Outcomes

Many studies reported improvements in selected mechanical properties following TiO_2_ incorporation [48,61,63,64,65,67,68,69,72,73,76,79,82,83,84,85,86,87,88,90,92,93]. However, the magnitude and consistency of these effects varied considerably among studies.

Most included studies reported an improvement in the compressive strength of glass ionomer cements following TiO_2_ incorporation, although the magnitude of the effect depended on both the concentration of TiO_2_ and the type of cement evaluated [61,64,65,67,69,72,73,82,86]. Enhanced compressive strength was consistently observed across studies investigating TiO_2_ concentrations ranging from 2 to 10 wt%, with several authors identifying 3–5 wt% as the optimal range for mechanical reinforcement [65,69,73,86]. Concentration-dependent increases were reported in multiple investigations [64,69], while excessively high TiO_2_ loadings were associated with reduced performance in some cases [73]. Improvements were observed in both conventional and restorative GIC formulations, although the response varied among different commercial products [64,86,87]. Notably, Shubha et al. [85] found significant improvements only at the highest concentration tested, whereas lower concentrations did not differ significantly from the control group. Despite the generally positive trend, Ivanisevic et al. reported a reduction in compressive strength and related mechanical properties following TiO_2_ incorporation, indicating that the reinforcing effect is not universal and may be influenced by factors such as particle concentration, dispersion, and cement composition [68].

The effect of TiO_2_ incorporation on the flexural strength of glass ionomer cements was less consistent than that observed for compressive strength. Several studies reported significant improvements in flexural strength following TiO_2_ addition [67,73,76,79,83,86,93]. In many cases, the greatest enhancement was observed at low to moderate nanoparticle concentrations, particularly around 3–5 wt%, whereas higher concentrations either produced no additional benefit or resulted in a decline in flexural performance [73,83,93]. Improvements were most evident in restorative GIC formulations, while some cement types showed little or no response to TiO_2_ modification [83,86]. However, not all studies demonstrated a positive effect. Fathi et al. and Kantovitz et al. [63,69] reported no statistically significant differences between TiO_2_-modified and conventional GICs, and Abozaid et al. [84] found higher mean values in TiO_2_-containing groups without statistically significant intergroup differences.

Surface hardness and microhardness were among the most consistently improved mechanical properties following TiO_2_ incorporation into glass ionomer cements. Most studies reported enhanced hardness values in TiO_2_-modified materials compared with conventional GICs [48,61,63,72,76,83,84,86,88]. The beneficial effect was generally concentration-dependent, with optimal results frequently observed at low to moderate TiO_2_ concentrations, particularly around 3–5 wt% [48,63,72,76,86]. Several authors reported that increasing TiO_2_ content beyond the optimal range led to reduced hardness, which was commonly attributed to nanoparticle agglomeration and impaired particle dispersion within the cement matrix [72,83]. However, some studies found the highest hardness values at greater concentrations, suggesting that the optimal loading may vary according to the specific GIC formulation [84]. Although the overall trend favored TiO_2_ modification, inconsistent findings were also reported. Ibrahim et al. and Hepdeniz et al. [67,92] observed only slight, statistically insignificant increases in hardness, while Panahandeh et al. [93] found reduced surface hardness across all TiO_2_ concentrations tested. Similarly, Shahpaska et al. demonstrated that the effect of TiO_2_ nanoparticles was material-dependent, with improvements observed in GC Fuji Triage but limited or negative effects reported for Ketac Universal [90].

#### 3.3.2. Physicochemical Properties Outcomes

Considering the physicochemical properties of GICs modified with titanium oxide, the studies included in this review mainly described water sorption and solubility, ion release and SEM microscopic evaluation of the surface of the innovative material samples [48,62,63,64,66,69,71,72,73,75,80,82,83,84,86,88,90,91,92].

The incorporation of TiO_2_ nanoparticles generally improved the resistance of glass ionomer cements to water-related degradation by reducing both water sorption and solubility [63,66,84,91]. Most studies demonstrated lower water uptake in TiO_2_-modified materials compared with conventional GICs, with the effect often becoming more pronounced as TiO_2_ concentration increased [66,84]. Similarly, reductions in solubility were consistently reported, suggesting enhanced structural stability and reduced susceptibility to dissolution in aqueous environments [63,84,91]. While Kantovitz et al. found no significant effect of TiO_2_ nanotubes on water sorption, they observed a concentration-dependent decrease in water solubility, with the greatest reduction at 5 wt% [91].

The influence of TiO_2_ incorporation on fluoride and ion release was variable across studies and appeared to depend on nanoparticle concentration, cement formulation, and evaluation period. Several investigations reported enhanced fluoride release from TiO_2_-modified GICs, particularly at higher nanoparticle concentrations and during the initial release phase [48,75]. Morales-Valenzuela et al. further demonstrated more sustained long-term fluoride release and improved fluoride recharge capacity in TiO_2_-containing materials [75]. In contrast, Wassel et al. observed a modest reduction in cumulative fluoride release compared with conventional GIC [82]. Studies evaluating broader ion-release profiles generally found limited effects of TiO_2_ addition on the release of most elements, although transient increases in fluoride and titanium release were reported during the early stages of storage [62,71]. Additionally, TiO_2_ incorporation was associated with a substantial reduction in aluminum ion release, which may have implications for the biocompatibility and chemical stability of the material [80]. Collectively, these findings suggest that TiO_2_ nanoparticles can modify the ion-release behavior of GICs, although the direction and magnitude of these changes are material-dependent.

Microscopic and spectroscopic analyses consistently demonstrated that TiO_2_ incorporation altered the microstructure of glass ionomer cements [64,72,73,86,87,88,90,91,92]. Most studies reported reduced porosity, fewer voids, and a lower incidence of microcracks in specimens containing low to moderate TiO_2_ concentrations, indicating a more compact and homogeneous microstructure [64,72,73,87]. These structural improvements were frequently associated with enhanced mechanical properties. However, higher nanoparticle concentrations often resulted in increased defect formation, including crack propagation and structural irregularities, which were commonly attributed to nanoparticle agglomeration [72,73]. Elemental and spectroscopic analyses confirmed successful incorporation of titanium into the cement matrix and revealed concentration-dependent changes in both elemental composition and chemical structure [86,91]. Nevertheless, the microstructural response varied among different GIC formulations, with some studies reporting increased surface cracking or material-specific differences in surface homogeneity following TiO_2_ addition [90,92]. The effect of TiO_2_ nanoparticles on surface roughness was less consistent. While some studies found no significant changes in surface topography after TiO_2_ incorporation [48,69], others reported smoother surfaces associated with a denser and more compact microstructure [88]. In contrast, increased roughness was observed in certain materials, particularly at higher nanoparticle concentrations, suggesting that excessive TiO_2_ loading may negatively affect surface uniformity [83].

#### 3.3.3. Biological Properties Outcomes

The available evidence suggests that TiO_2_ incorporation generally maintains or improves the biocompatibility of glass ionomer cements, although the magnitude of this effect appears to depend on the cement formulation and nanoparticle characteristics [48,70,74,75,77]. Several studies reported preserved or enhanced cell viability, normal cell morphology, and sustained cellular proliferation in TiO_2_-modified materials [48,74,77]. In addition, TiO_2_-containing GICs demonstrated favorable biological effects, including increased extracellular matrix production, reduced genotoxicity, and modulation of inflammatory responses. Specifically, TiO_2_ incorporation was associated with decreased expression of pro-inflammatory mediators such as IL-1β, IL-6, TNF-α, and VEGF, while maintaining stable levels of the anti-inflammatory cytokine IL-10 [74]. Similarly, TiO_2_ nanotubes were shown to attenuate lipopolysaccharide-induced upregulation of IL-1β, IL-6, IL-10, VEGF, and TNF-α, indicating a potential immunomodulatory effect [77]. However, not all studies reported positive outcomes. Morales-Valenzuela et al. observed variability among different commercial cements, with some TiO_2_-modified formulations showing no cytotoxicity and others demonstrating moderate toxicity [75]. Similarly, Cvjeticanin et al. reported generally low cytocompatibility across all tested materials, with lower cell viability in TiO_2_-modified Ketac-based cements than in Fuji-based cements [62]. Garcia-Contreras et al. (2014) found that TiO_2_ incorporation did not substantially alter the cytotoxic profile of GICs, although certain formulations induced ultrastructural cellular alterations, including irregular cell membranes and cytoplasmic vacuolization, and significantly increased PGE_2_ production, indicating a potential pro-inflammatory response [89].

The effect of TiO_2_ incorporation on the antibacterial properties of glass ionomer cements was investigated extensively, with most studies reporting enhanced antimicrobial activity compared with unmodified GICs [65,71,81,82,85,86]. Improved antibacterial performance was commonly observed at low to moderate nanoparticle concentrations, particularly between 3 and 5 wt%, whereas higher concentrations did not necessarily provide additional benefits and, in some cases, reduced antibacterial effectiveness [71,81]. Several studies demonstrated larger bacterial inhibition zones, reduced bacterial viability, alterations in bacterial morphology, and modulation of bacterial virulence-associated gene expression following TiO_2_ incorporation [65,81,82]. Nevertheless, the antibacterial effect was not consistently observed across all studies or cement formulations. One study reported reduced biofilm density on TiO_2_-modified specimens, although quantitative microbiological analyses did not reveal significant differences compared with conventional GIC [67]. Similarly, de Gois Sena et al. found no significant improvement in antibacterial activity against *Lactobacillus acidophilus* [78].

#### 3.3.4. Other Properties Outcomes

Several studies evaluated additional properties of TiO_2_-modified GICs, including optical characteristics, radiopacity, handling properties, and material aging. TiO_2_ incorporation generally increased radiopacity and altered color parameters in a concentration-dependent manner, although adhesion to dentin remained unaffected [77,91]. The influence on working and setting times was inconsistent, with most studies reporting minimal or no clinically relevant changes, while high TiO_2_ concentrations occasionally prolonged or shortened setting times depending on the material formulation [80,85,91]. Limited evidence also suggests that TiO_2_-modified GICs continue to undergo microstructural changes during aging, indicating ongoing interactions between nanoparticles and the cement matrix over time [90] (see Table 2).

### 3.4. Quality Assessment

According to the QUIN tool, thirty-one of the included studies were categorized as having medium risk of bias scoring either 58.3% [61,62,63,64,65,66,67,68,70,72,73,74,75,76,79,84,86,87,89,90,92,93] or 66.67% [48,69,71,77,78,82,83,85,88]. Three studies scored over 70% having a low risk of bias [80,81,91] (see Supplementary Table S3). The methodological domains most frequently given low scores were the absence of blinding, insufficient reporting of randomization procedures, and inadequate descriptions of the personnel performing the experimental procedures and outcome assessments. These limitations were common among the included in vitro studies and contributed substantially to the predominance of medium risk of bias classifications.

## 4. Discussion

This systematic review synthesized the available in vitro evidence regarding the effect of titanium dioxide (TiO_2_) incorporation into conventional glass ionomer cements (GICs). The analysis of the included studies suggests that TiO_2_ incorporation at concentrations between 3 and 5 wt% frequently improved selected mechanical, physicochemical, biological and antimicrobial properties under in vitro conditions while preserving the fundamental advantages of GICs. However, these effects are not universal and vary according to nanoparticle concentration, morphology, particle dispersion, and the type of GIC used [48,61,62,63,64,65,66,67,68,69,70,71,72,73,74,75,76,77,78,79,80,81,82,83,84,85,86,87,88,89,90,91,92,93].

The current synthesis indicates that TiO_2_ incorporation frequently improves the mechanical performance of conventional GICs, particularly compressive strength, surface hardness and flexural strength [48,61,63,64,67,68,69,72,73,76,79,82,83,84,85,86,88,90,92,93]. The majority of studies reporting improvements identified 3–5 wt% as the most favorable concentration range, which represents the optimal proportion for reinforcement of the glass ionomer cements [48,63,64,69,72,73,86,90,93]. At these concentrations, TiO_2_ nanoparticles can be more uniformly dispersed within the cement structure and occupy interstitial spaces between larger glass particles. This improves particle packing and contributes to a denser and more homogeneous microstructure, reducing internal voids and limiting crack initiation and propagation under mechanical stress [46,94]. Furthermore, the increased surface area of well-dispersed nanoparticles may enhance particle–matrix interactions, improving structural cohesion and stress distribution throughout the material [95,96]. However, the relationship between TiO_2_ concentration and mechanical performance was not consistent across all studies. While concentrations above 5 wt%, particularly 10 wt%, were frequently associated with increased porosity, microcrack formation and deterioration of mechanical properties [72,93,97], Gjorgievska et al. and Shubha et al. reported significant improvements even at 10 wt% TiO_2_ loading [85,87]. These discrepancies may be explained by variations in cement composition, nanoparticle properties, and experimental methods. At higher loadings, nanoparticle agglomeration can occur due to high surface energy, creating defects and stress concentration sites within the matrix [98,99]. In addition, higher nanoparticle loadings require greater wetting and dispersion capacity from the liquid component of the cement. If complete wetting cannot be achieved, poorly bonded regions may develop around nanoparticle clusters, weakening the material and increasing structural heterogeneity [100,101,102]. Moreover, excessive nanoparticle incorporation may interfere with the acid-base setting reaction of GICs and disrupt matrix formation [19,103]. Consequently, although moderate TiO_2_ concentrations generally enhance matrix reinforcement and mechanical stability, the optimal concentration appears to depend on nanoparticle morphology, dispersion quality and the specific commercial GIC formulation employed [104,105].

Conventional glass ionomer cements are characterized by relatively high water sorption and solubility, which constitute important limitations affecting their long-term clinical performance [106]. Excessive water uptake during the early stages of maturation may compromise structural integrity, accelerate surface degradation, and negatively influence the mechanical stability of the material [107,108]. Most studies included in this review demonstrated that the incorporation of titanium oxide into the GIC structure significantly reduces this unfavorable phenomenon, thereby improving the cement’s resistance to hydrolytic degradation [63,66,84,91]. This effect is likely related to microstructural modifications induced by TiO_2_, including reduced porosity and a more homogeneous structure that limits the penetration of aqueous media through interconnected voids [109,110]. Consequently, dissolution processes become limited, leading to improved dimensional stability and greater resistance to hydrolytic degradation in the moist oral environment. Within the limitations of the included in vitro studies, these improvements suggest that TiO_2_-modified glass ionomer cements may demonstrate enhanced durability and stability-related properties compared with conventional formulations. However, their long-term clinical performance remains to be confirmed in clinical studies [111,112].

Several studies reported increased fluoride release and recharge capacity following TiO_2_ incorporation [48,62,64,71,75,78,82]. Moreover, the studies by Cibim et al. and Morales-Valenzuela et al. these materials showed a more stable fluoride release profile over time [75,113]. Additionally, these modified cements showed superior fluoride recharge capacity, meaning they were able to reuptake fluoride from external sources and release it again more effectively [75,114,115,116,117]. The explanation for this phenomenon again lies in the microstructure of the material [118,119]. The incorporation of titanium oxide strengthens the cement structure, but the microchannels and porosities responsible for ion diffusion are preserved. These channels are essential pathways for fluoride-ion transport and therefore their preservation may maintain ion mobility within the material [75,120,121]. Meanwhile, the presence of TiO_2_ can stabilize the structure while promoting more controlled and long-lasting ion exchange rather than rapid depletion [75,122].

Previous studies have shown that conventional glass ionomer cements are characterized by good biocompatibility, supporting the growth of various human and animal cell lines [123,124,125]. Similar behavior is observed on titanium surfaces, where a stable TiO_2_ layer limits ion release and supports cell adhesion, partly due to nanotubular structures that increase surface area and enhance protein adsorption and cell attachment [126,127,128]. Consistently, studies included in this systematic review indicate that incorporating titanium dioxide into GIC maintains satisfactory biocompatibility and may even improve it [48,74,77]. Some authors additionally report reduced expression of pro-inflammatory mediators, suggesting that TiO_2_ incorporation may attenuate inflammation at the tooth–restoration interface and modulate angiogenic activity, as reflected by decreased VEGF expression [74,77]. Together with reduced levels of IL-1β, IL-6, and TNF-α, this suggests that TiO_2_ incorporation may promote a more biologically favorable response in the experimental models evaluated [129,130].

Another important finding of the present review was the enhanced antibacterial activity of TiO_2_-modified cements. Increased antibacterial activity after the incorporation of titanium dioxide into GIC was noticed for cariogenic bacteria *Streptococcus mutans* and *Lactobacillus acidophilus*, important in the aspect of considerations regarding restorative materials in dentistry [78,81,82,85]. Hamid et al. [65], reported that culturing these bacterial strains on modified samples resulted in reduced bacterial viability and inhibition of biofilm formation. In addition to easily obtainable observations regarding the morphology and number of colonies, genes responsible for bacterial virulence, such as *covR* and *vicR*, whose expression was reduced, were also assessed [81]. This effect is largely attributed to the oxidative properties of TiO_2_, which generate reactive oxygen species (e.g., hydroxyl radicals and superoxide ions) that damage bacterial membranes, proteins, and nucleic acids [131,132,133,134]. Additional antibacterial benefits arise from increased fluoride release and smoother cement surfaces, which further limit bacterial adhesion and biofilm accumulation [135,136,137,138].

Although many studies reported beneficial effects of TiO_2_ incorporation, the available evidence was not uniformly positive. Significant improvements were most consistently observed for compressive strength and surface microhardness, particularly at concentrations between 3 and 5 wt%. In contrast, findings regarding flexural strength, ion release, antibacterial activity, and cytocompatibility were more variable. Several studies reported non-significant differences compared with controls, whereas others demonstrated unfavorable outcomes. These discrepancies may be explained by differences in TiO_2_ concentration, particle morphology, synthesis route, particle dispersion, testing protocols, storage conditions, and the commercial GIC formulation used. Therefore, the reported effects should be interpreted within the context of substantial methodological and material heterogeneity among the included studies.

The findings of this review suggest that the incorporation of titanium dioxide into conventional glass ionomer cement may enhance several properties of the material, including its mechanical, physicochemical, biological, and antimicrobial characteristics. Nevertheless, these results should be interpreted with caution due to several limitations of the available evidence. All included studies were conducted in vitro. Although laboratory studies are essential for initial material assessment, they cannot fully replicate the complexity of the oral environment, including saliva composition, biofilm formation, masticatory forces, thermal cycling, pH fluctuations, and patient-related factors. Therefore, the favorable outcomes observed under controlled conditions may not directly translate into clinical effectiveness. Considerable methodological heterogeneity was identified across studies, including differences in TiO_2_ concentration, particle size, synthesis and characterization methods, incorporation protocols, GIC formulations, and testing procedures. This variability limits direct comparison between studies and may partly explain inconsistencies in reported outcomes, while also complicating the identification of an optimal formulation. Most studies focused on short-term evaluations, with limited data on long-term aging, degradation resistance, sustained ion release, color stability, and durability under simulated oral conditions. As a result, the long-term performance and stability of TiO_2_-modified GICs remain insufficiently established. In addition, only English-language studies were included, which may introduce language bias and limit the comprehensiveness of the review. Future research should prioritize standardized experimental protocols to enable more reliable comparisons and clearer identification of optimal TiO_2_ formulations. Long-term in vitro and in situ studies are needed to better simulate oral conditions, while well-designed clinical trials are essential to determine whether laboratory improvements translate into meaningful clinical benefits.

## 5. Conclusions

A comprehensive review of the 34 included studies suggests that the incorporation of titanium dioxide (TiO_2_) into conventional glass ionomer cements may enhance selected mechanical, physicochemical, biological, and antibacterial properties. However, these findings should be interpreted with caution due to substantial heterogeneity among the included studies regarding TiO_2_ form, particle size, synthesis method, concentration, glass ionomer cement formulation, and assessed outcomes. The most favorable effects were generally observed at TiO_2_ concentrations of approximately 3–5 wt%, whereas higher concentrations were sometimes associated with nanoparticle agglomeration and less favorable or reduced material performance. Overall, the available evidence indicates that TiO_2_ may be a promising additive for selected improvements in conventional glass ionomer cements; however, its effects appear to be strongly concentration- and formulation-dependent. Further standardized long-term in vitro, in situ, and well-designed clinical studies are needed to confirm whether these laboratory findings translate into clinically meaningful benefits under oral cavity conditions.

## Figures and Tables

**Table 1:** Search syntax used for identifying relevant publications.

Source	Search Term
PubMed	(glass ionomer [All Fields]) AND (titanium oxide [All Fields] OR TiO_2_ [All Fields] OR titanium dioxide [All Fields] OR titanium nanotubes [All Fields] OR titanium nanoparticles [All Fields])
Scopus	TITLE-ABS-KEY(glass ionomer) AND (titanium dioxide OR TiO_2_ OR titanium oxide OR titanium nanotubes OR titanium nanoparticles)
Embase	(‘glass ionomer’:ab,ti) AND (‘titanium dioxide’:ab,ti OR TiO_2_:ab,ti OR ‘titanium oxide’:ab,ti OR ‘titanium nanotubes’:ab,ti OR ‘titanium nanoparticles’:ab,ti)
Web of Science	((TS = glass ionomer) AND (((((TS = titanium dioxide) OR (TS = TiO_2_)) OR (TS = titanium oxide)) OR (TS = titanium nanotubes)) OR (TS = titanium nanoparticles)))
WorldCat	(glass ionomer) AND (titanium dioxide OR TiO_2_ OR titanium oxide OR titanium nanotubes OR titanium nanoparticles)

**Table 2:** Detailed Characteristics of Included Studies.

Study	Type of GIC	Amount/ Concentration of Added TiO_2_ (wt%)	Mechanical Properties Outcomes	Physicochemical/Structural Properties Outcomes	Biological Properties Outcomes	Other Outcomes
Assery et al. (2020) [61]	Harvard Ionoglas Cem, (Harvard Dental International GmbH, Hoppegarten, Germany)	5 wt% TiO_2_	**Compressive Strength:** the highest in GIC + TiO_2_ group (154.2 ± 2.4 MPa) **Diametral Tensile Strength:** the highest in GIC + TiO_2_ group (13.2 ± 0.5 MPa) **Flexural Strength**: the highest in GIC + Ag group, GIC + TiO_2_ showed lower values, but higher than the control group **Hardness:** the highest for GIC + TiO_2_ nanoparticles (90.4 ± 1.1)	n/a	n/a	n/a
Cibim et al. (2017) [48]	Ketac Molar Easymix (3M/ESPE, Maplewood, MN, USA)	3 wt% TiO_2_; 5 wt% TiO_2_; 7 wt% TiO_2_	**Surface Hardness:** GIC + 5 wt% TiO_2_ showed the highest values (118.25 ± 4.21) compared to control and other concentrations	**Fluoride release**: in both demineralizing and remineralizing solutions the highest fluoride burst was observed during first 48 h and then gradually decreased; higher concentrations of released fluoride were observed in GIC+ 5 wt% TiO_2_ and GIC + 7 wt% compared to control and to GIC + 3 wt% **Surface Roughness:** TiO_2_ incorporation did not alter surface topography **EDS Analysis**: All groups showed dominance of calcium and phosphorus; titanium was only detected in 5% and 7% TiO_2_ groups, confirming incorporation.	**Cell Viability (MTT Assay)**: GIC+ 5% TiO_2_ showed the highest cell viability **SEM analysis:** TiO_2_-NT incorporation did not impair cell adhesion or growth. **ECM Production:** collagenous ECM—increased over time for GIC + 5% TiO_2_ noncollagenous ECM—increased over time for GIC + 3% TiO_2_.	n/a
Cvjeticanin et al. (2024) [62]	Fuji IX (GC Corp., Tokyo, Japan), Ketac Molar EasyMix (3 M/ESPE, Deutschland GmbH, Neuss, Germany).	5 wt% TiO_2_	n/a	**Ion release:** The TiO_2_-NPs addition did not increase the release of ions except for titanium and fluoride in the initial phase both in case of Fuji IX and Ketac Molar EasyMix modification	**Cell viability:** All GIC samples showed low cytocompatibility, with cell viability below 50%; Ketac-based cements, especially those modified with TiO_2_ or Mg-doped hydroxyapatite, induced significantly lower cell viability than Fuji-based cements.	n/a
Fathi et al. (2022) [63]	Conventional glass ionomer cement powder (Cavex, Ofterdingen, Germany)	3 wt% TiO_2_ 5 wt% TiO_2_	**Surface Microhardness:** significant increase in groups doped with TiO_2_, especially in samples with 5 wt% TiO_2_ compared to the control **Flexural Strength:** No significant differences between groups were noted	**Water solubility:** significant decrease in modified groups compared to control **Water sorption:** significant decrease in modified groups compared to control	n/a	n/a
Gjorgievska et al. (2020) [64]	ChemFil Rock (Dentsply DeTrey, Konstanz, Germany); GC Equia Fil (GC Europe N.V., Leuven, Belgium)	2 wt% TiO_2_; 5 wt% TiO_2_; 10 wt% TiO_2_	**Compressive Strength:** GC Equia Fil: after one week 5 wt% loading tended to show lower compressive strength compared to 2 and 10 wt% ChemFil Rock: all TiO_2_ loadings (2, 5, 10 wt%) produced stronger samples than control; the strength increased with concentration with 10% loading showing the highest compressive strength after 1 week.	**SEM analysis:** TiO_2_ incorporation reduced porosity, fracture lines propagated more through the matrix rather than along voids, the effect was most evident with 10% TiO_2_ after 1 week for GC Equia Fil; for ChemFil Rock, the best effect was observed with 5% TiO_2_ incorporation **Ion release:** the addition of TiO_2_ alters ionic behavior indirectly, even if Ti release itself is minimal	n/a	n/a
Garcia-Contreras et al. [86] (2015)	n/a	3 wt% TiO_2_ 5 wt% TiO_2_	**Surface Microhardness:** doping the restoration cement with 3 and 5 wt% TiO_2_ significantly increased microhardness compared to conventional cement; in case of core shade base cement and base cement a decrease in microhardness was observed **Flexural strength:** Significant improvement in restorative GIC enriched with 3 and 5%wt TiO_2_; no improvement in core shade and base cement groups; **Compressive strength:** Significant improvement in restorative GIC enriched with 3 and 5%wt TiO_2_; core shade cement—improvement only in group doped with 5 wt% TiO_2_; base cement—no improvement **Shear bond strength:** Not significant increase in shear bond strength to enamel for the core shade cement containing 5 wt%TiO_2_ and FX-II containing 3 and 5 wt% TiO_2_-NPs	**SEM analysis:** no major topographical differences between groups **EDS analysis**: titanium detected in modified groups; increase of oxygen and decrease of strontium concentration by incorporating TiO_2_	**Antibacterial activity:** incorporation of 3 and 5%wt TiO_2_ to conventional restorative GIC significantly increased antibacterial properties; no antibacterial effect in core shade and base cement	n/a
Gjorgievska et al. [87] (2015)	ChemFil Rock (Dentsply DeTrey, Konstanz, Germany); GC Equia Fil (GC Europe N.V., Leuven, Belgium)	10 wt% TiO_2_	**Compressive Strength:** the addition of 10 wt% TiO_2_ to conventional ChemFil Rock and GC Equia Fil significantly increased the compressive strength from 33.0 ± 9.9 MPa to 47.2 ± 5.3 MPa and from 32.3 ± 2.4 MPa to 42.1 ± 5.3 MPa respectively	**SEM analysis:** compared to unmodified GIC the incorporation of TIO_2_ reduces air voids, which if appear are shallower, also fewer cracks were observed. **EDX analysis:** Ti was detected however there was little Ti migration to matrix.	n/a	n/a
Hamid et al. (2019) [65]	GC Fuji IX Gold Label (GC, Isnapur, India)	3 wt% TiO_2_	**Compressive Strength:** 3 wt% TiO_2_ incorporation to conventional GIC showed increased compressive strength—140.0287 (±9.07569) vs. 172.5483 (±14.8844)	n/a	**Antibacterial activity:** GIC modified with 3 wt% TiO_2_-NPs exhibited greater inhibition zone 21.16667 (±3.563281) than the conventional GIC 15.75 (±2.301185)	n/a
Hussein et al. (2022) [66]	Kromoglass 2 (LASCOD Spa-Via L.Longo, 18 50019 Sesto Fiorentino (Firenze), Italy).	10 wt% TiO_2_	n/a	**Water sorption:** TiO_2_ modified glass ionomer cement showed significantly lower water sorption compared to the conventional type, particularly in artificial saliva at 1 week and in alcohol mouthwash at 24 h. Over time, a significant gradual decrease in sorption was observed for the TiO_2_-modified cement in all storage solutions, with the lowest values recorded at 1 month. **Water solubility:** TiO_2_ modification reduced early solubility; solubility was highest in alcohol at 1 month, while in artificial saliva and alcohol-free mouthwash the peak occurred at 1 week	n/a	n/a
Ibrahim et al. (2017) [67]	GC Gold Label Glass Ionomer High Strength Posterior Restorative (GC Corporation, Tokyo, Japan)	3 wt% TiO_2_	**Flexural strength:** TiO_2_-GIC exhibited higher flexural strength compared to unmodified GIC **Compressive strength:** TiO_2_-GIC exhibited higher compressive strength compared to unmodified GIC **Surface hardness:** TiO_2_-GIC exhibited higher surface hardness compared to unmodified GIC, but the difference was not statistically significant	n/a	**SEM analysis:** in case of TiO_2_-GIC the observed biofilm on the disk was thinner and less dense on non-modified GIC disks **Confocal Microscopy:** the 3 wt% TiO_2_-GIC showed slight increase in dead bacteria compared to unmodified GIC. **CFU counts:** the GIC disks modified with TiO_2_ exhibited lower CFU counts but compared to conventional GIC the difference was not statistically significant **MTS assay:** GIC modified with TiO_2_ exhibited lower absorbance compared to conventional GIC the difference was not statistically significant	n/a
Ivanišević et al. (2021) [68]	Fuji IX GP Extra (GC Corporation, Tokyo, Japan)	3 wt% TiO_2_	**Compressive strength:** TiO_2_-GIC exhibited lower compressive strength compared to unmodified GIC. **Breaking strength:** TiO_2_-GIC exhibited lower breaking strength compared to unmodified GIC. **Compressive modulus:** TiO_2_-GIC exhibited lower compressive modulus compared to unmodified GIC.	n/a	n/a	n/a
Kantovitz et al. (2020) [69]	Ketac Molar EasyMixTM-3M/ESPE, Maplewood, MN, USA).	3, 5 and 7 wt% TiO_2_	**Compressive strength:** 5% TiO_2_-GIC exhibited the highest compressive strength compared to other groups **Flexural strength:** the difference was not statistically significant **Microshear bond strength and failure mode to dentin:** The 5% TiO_2_-NT group had higher MSBS values than the 7% group with no significant differences compared to the 3% and control groups. **Weight loss before and after brushing simulation:** TiO_2_-NT significantly reduced the matrix weight loss of GIC, independent of its concentration	**Surface roughness:** the difference was not statistically significant	n/a	n/a
Laiteerapong et al. (2018) [70]	GC Gold Label 9 HS Posterior EXTRA, (GC Corporation, Tokyo, Japan)	10 wt% TiO_2_	n/a	n/a	**Cytotoxicity:** showed a comparable cytotoxic effect for TiO_2_NPs-GIC and GIC, whereas TiO_2_MPs-GIC tended to exhibit lower cytotoxicity **Genotoxicity:** At both concentrations, all cement groups showed significantly fewer mean foci than the positive control, with no differences between cement types. However, TiO_2_-modified GICs exhibited a higher percentage of foci-free cells than the negative control and conventional GIC, suggesting reduced genotoxicity.	n/a
Mahendra et al. (2023) [71]	GC Fuji II, Pyrax Polymers, (Roorkee, India)	3 and 5 wt% TiO_2_	n/a	**Ion release:** The 5% TiO_2_ group consistently exhibited higher titanium ion release than the 3% group, with the highest levels observed during the first two months and peaking at month two.	**Antibacterial activity:** 5% TiO_2_-GIC showed significantly higher antibacterial activity than 3% TiO_2_-GIC	n/a
Mansoor et al. (2024) [72]	GC Fuji Universal Gold Label 2	3, 5, 7, 10 wt% TiO_2_	**Microhardness strength:** The Vickers microhardness analysis exhibited the highest microhardness strength in 5% TiO_2_-GIC. Beyond this, a linear decrease in microhardness was seen with higher TiO_2_ concentrations in groups 7% TiO_2_ GIC and 10% TiO_2_ GIC.	**SEM analysis:** Non-modified GIC had the most cracks due to low hardness. 5 wt% TiO_2_-GIC resulted in minimum cracks and maximum microhardness compared to other groups indicating optimal performance. Higher TiO_2_ wt% resulted in reduced hardness and increased crack formation.	n/a	n/a
Mansoor et al. (2022) [73]	n/a	3, 5, 7, 10 wt% TiO_2_	**Flexural strength:** compared to unmodified cement (16.11MPa), the flexural strength increased with TiO_2_ incorporation up to 5% (26.39 MPa), then decreased at higher concentrations. **Compressive strength:** The best compressive strength was observed in GIC modified with 5 wt% TiO_2_ (15.51 MPa), which was almost a double compared to conventional GIC (7.63 MPa). The properties decreased with higher TiO_2_ additive concentration.	**SEM analysis:** Conventional GIC showed high porosity and micro-cracks; 3–5% TiO_2_ demonstrated reduced porosity and cracks, while 7–10% TiO_2_ increased defects again.	n/a	n/a
Meyer et al. (2025) [74]	Ketac Molar EasyMixTM-3 M/ESPE, (Maplewood, MN, USA);	3, 5 and 7 wt% TiO_2_	n/a	n/a	**Cell viability:** TiO_2_ incorporation maintained or slightly improved cell viability compared with GIC alone, showing no cytotoxic effects. **Cell proliferation:** TiO_2_ reinforced GICs showed normal or slightly increased proliferation of MDPC-23 cells over 72 h **Inflammatory response:** TiO_2_ reduced proinflammatory markers (IL-1β, IL-6, TNF-α, VEGF) and stabilized anti-inflammatory IL-10 expression compared with GIC alone.	n/a
Morales-Valenzuela et al. (2022) [75]	Fuji IX Extra (GC, Kyoto, Japan), Ketac Molar (3M ESPE, Maplewood, MN, USA), Ionofill Molar (Voco, Cuxhaven, Germany), Fuji IX (GC, Kyoto, Japan)	3 wt% TiO_2_	n/a	**Fluoride release:** All materials released fluoride over time up to 300 days. TiO_2_ reinforced materials released up to 3x more fluoride than control cements, and the release maintained longer than in case of control specimens Modified cements presented a more stable and sustained fluoride release profile In terms of fluoride recharge, experimental cements exhibited better performance of fluoride re-release after applying fluoride gel compared to the control group.	**Cytotoxity**: Both Fuji IX Extra and Ketac Molar modified with TiO_2_ turned out to be safe and exhibited no cytotoxicity while Ionofil and Fuji IX modified with TiO_2_ showed moderate toxicity.	n/a
Ramić et al. (2024) [76]	Fuji IX GP (GC International, Tokyo, Japan) Ketac Molar EasyMix (3M ESPE, Maplewood, MN, USA)	5 wt% TiO_2_	**Flexural strength:** Fuji IX + 5% TiO_2_ and Ketac Molar + 5% TiO_2_ exhibited higher values compared to the control **Fracture Toughness (KIC):** Ketac Molar + 5% TiO_2_: significant increase Fuji IX: no changes **Microhardness:** Fuji IX: significant increase with 5% TiO_2_	n/a	n/a	n/a
Rangel-Coelho et al. (2024) [77]	Ketac Molar EasyMix (3M ESPE)	3, 5 and 7 wt% TiO_2_	n/a	n/a	**Cell proliferation**: increased over time at 24/48/72 h regardless of TiO_2_-NT or LPS; **Mitochondrial metabolism (MTT):** increased over time; TiO_2_-NT did not alter metabolic activity **Cell morphology:** not affected **Cytokine secretion:** TiO_2_-NT reversed LPS-induced upregulation of IL-1β, IL-6, IL-10, VEGF, and TNF-α at 12 h **Gene expression:** TiO_2_-NT reversed GIC-alone transcript levels; IL-1β elevated by LPS across all groups; GIC alone promoted VEGF expression at 72 and 120 h, reversed by 5% and 7% TiO_2_-NT	**Adhesion to dentin:** not affected by TiO_2_-NT **Color stability:** color opacity improved with TiO_2_-NT **Radiopacity:** improved with TiO_2_-NT
de Gois Sena et al. (2024) [78]	Ketac Molar EasyMix (3M/ESPE, Maplewood, MN, USA)	3, 5, and 7 wt% TiO_2_	n/a	n/a	**Inhibition zone:** no significant difference between GIC and any TiO_2_ group at any time point; inhibition zone increased from day 1 to days 3 and 7 within all groups **Cell morphology:** TiO_2_ incorporation did not alter *L. acidophilus* morphology	n/a
Showkat et al. (2023) [79]	GC Corporation, Tokyo, Japan	3 wt% TiO_2_	**Flexural Strength** - Group I (Control): 5.26 ± 1.03 MPa - Group 3% TiO_2_: 27.81 ± 3.50 MPa	n/a	n/a	n/a
da Silva Morais et al. (2022) [80]	Ketac Molar EasyMix (3M/ESPE, Maplewood, MN, USA)	5 wt% TiO_2_	n/a	**EDS/SEM surface composition:** Overall elemental composition comparable between groups with and without pH-cycling. Ti was not detected by EDS in TiO_2_-NT group. Sodium was the only element significantly altered at baseline. Phosphorus and lanthanum increased with pH-cycling in both groups. TiO_2_ incorporation reduced Al release ~60% on days 1–5 and 100% by day 7	n/a	**Initial working time:** Control group: 321.4 ± 3.4 s Ketac Molar + 5% TiO_2_: 319.9 ± 7.1 s
de Souza Araujo et al. (2021) [81]	Ketac Molar EasyMix (3M/ESPE, Maplewood, MN, USA)	3, 5, and 7 wt% TiO_2_	n/a	n/a	**Inhibition zone:** GIC + 5% TiO_2_ produced the largest inhibition zones, whereas GIC + 7% TiO_2_ showed the lowest antibacterial activity. **Viability assay:** TiO_2_-modified GICs reduced bacterial viability. GIC + 3% TiO_2_ and GIC + 5% TiO_2_ were most effective at day 1, while GIC + 7% TiO_2_ showed a progressive reduction, reaching the strongest effect at day 7. **SEM analysis:** GIC + 3% TiO_2_ and GIC + 5% TiO_2_ altered bacterial morphology, inducing rod-shaped cells and linear arrangements. **Gene expression:** All TiO_2_ concentrations downregulated *covR* at 24 h, with the greatest effect at 3% TiO_2_. *vicR* expression was significantly reduced only in the GIC + 3% TiO_2_ group at 72 h.	n/a
Wassel et al. (2022) [82]	Riva self-cure GIC (SDI, Bayswater, Australia)	5 wt% TiO_2_	**Compressive strength:** - Control group: 136.48 ± 13.40 MPa - Group Ti: 166.31 ± 15.08 MPa	**Fluoride ion release:** at 24 h: Control 0.16 > Ti 0.14 µg/mm^2^ at 14 days: control 0.21 > Ti 0.20 µg/mm^2^ at 28 days: Control 0.19 > Ti 0.13 µg/mm^2^ Cumulative at 28 days: Control 0.560 > Ti 0.470 µg/mm^2^	***S. mutans* inhibition zone results (mm):** Control group: 0.16 ± 0.012 mm Group Ti: 28.50 ± 5.52 mm—significant difference in reference to the control group	n/a
Karamüftüoğlu et al. (2026) [83]	Ketac Molar Easymix) and Ketac Cem Radiopaque (3M ESPE Dental Products, St. Paul, MN, USA)	1, 3, and 5 wt% TiO_2_	**Flexural strength:** For Ketac Molar Easymix, strength improved mainly at low nanoparticle levels (1–3%), while Ketac Cem Radiopaque showed no significant change. **Microhardness:** Hardness increased at low TiO_2_ concentrations (especially 1–3%) in both materials, but dropped at higher loading (5%), likely due to nanoparticle agglomeration.	**Surface roughness:** Adding TiO_2_-NPs made both cements progressively rougher, with the highest roughness consistently seen at 5% concentration (and significant differences between groups).	n/a	n/a
Abozaid et al. (2026) [84]	Fuji IX GP (GC Corporation, Tokyo, Japan)	5 and 10 wt% TiO_2_	**Flexural strength:** The 10% TiO_2_ group showed the highest mean strength, but overall differences between groups were not statistically significant. **Microhardness:** The 10% TiO_2_ group performed best, showing significantly higher hardness than both the control and 5% group.	**Water sorption:** Water sorption decreased as TiO_2_ content increased, with the lowest values in the 10% group and the highest in the control. **Solubility:** TiO_2_ addition reduced solubility, with significant differences compared to the control group.	n/a	n/a
Shubha et al. (2025) [85]	Fuji II, (GC Corporation, Tokyo, Japan)	50 mg/g = 5 and 100 mg/g = 10 wt% TiO_2_	**Compressive strength:** Only the 10% groups showed a significant increase in compressive strength, whereas 50 mg/g groups did not differ from the control.	n/a	**Antimicrobial activity:** TiO_2_ addition led to only a slight but statistically significant reduction in *S. mutans* and *L. acidophilus*, mainly at higher concentration, with overall modest antibacterial effects. **Antioxidant activity:** All formulations showed very low antioxidant activity, with TiO_2_ providing only a minimal, not significant increase compared to the control.	**Setting time:** 10% TiO_2_ loading significantly shortened the setting time of the material, while lower concentrations showed no significant change.
Ganesh et al. (2026) [88]	GC Corporation, Tokyo, Japan	5 wt% TiO_2_	**Microhardness:** highest in TiO_2_-GIC group (78.42 ± 3.15 VHN) **Compressive strength:** highest in TiO_2_-GIC group (165.42 ± 8.36 MPa) **Surface roughness:** Lowest in TiO_2_-GIC group (0.82 ± 0.07 µm).	**SEM analysis:** TiO_2_-GIC exhibited a dense, compact microstructure with uniformly distributed nanoparticles, indicating good dispersion within the glass matrix.	**Antibacterial activity:** TiO_2_-GIC demonstrated intermediate antibacterial activity, showing inhibition zones of 14.52 ± 1.08 mm against *S. mutans* and 13.64 ± 1.12 mm against *L. acidophilus*. Conventional GIC exhibited the lowest antibacterial performance, with inhibition zones of 9.76 ± 0.95 mm against *S. mutans* and 8.84 ± 0.88 against *L. acidophilus*	n/a
Garcia-Contares et al. (2014) [89]	Base cement, Core shade cement and FX-II (Shofu Dental Corp. Kyoto, Japan)	3, 5 wt% TiO_2_	n/a	n/a	**Cytotoxicity:** All glass ionomer cements, with and without TiO_2_ nanoparticles, showed significantly greater cytotoxicity toward oral cancer cells than normal oral cells. The addition of TiO_2_ nanoparticles did not markedly alter this pattern. **PGE_2_ production:** FX-II and FX-II modified with 3% TiO_2_ nanoparticles significantly increased PGE_2_ production in normal oral cells, with a stronger effect in HGF cells. In combination with IL-1β, both materials showed a synergistic enhancement of PGE_2_ production. **Cell morphology:** Exposure to FX-II, with or without 3% TiO_2_ nanoparticles, induced ultrastructural changes including irregular cell membranes and cytoplasmic vacuolization.	n/a
Shahpaska et al. (2026) [90]	GC Fuji TRIAGE (GC Corporation, Tokyo, Japan) Ketac Universal (3M/ESPE, Maplewood, MN, USA)	2, 5, and 10 wt% TiO_2_	**Microhardness:** In GC Fuji TRIAGE, TiO_2_-NPs generally increased microhardness, although TiO_2_ showed limited long-term effects. In Ketac Universal, nanoparticle addition generally reduced or did not significantly improve microhardness, with higher concentrations producing more pronounced reductions.	**AFM analysis:** In Fuji TRIAGE, nanoparticle incorporation generally increased surface irregularity and the density of surface peaks and valleys, except for 10 wt% TiO_2_, which maintained a morphology similar to the control. In Ketac Universal, lower concentrations of TiO_2_ (2–5 wt%) produced a denser and more homogeneous surface	n/a	**Aging of the material:** Surface changes observed after 21 days indicated that the interaction between nanoparticles and the cement matrix evolved over time.
Kantovitz et al. (2023) [91]	Ketac Molar EasyMix (3M/ESPE, Maplewood, MN, USA)	3, 5 and 7 wt% TiO_2_	n/a	**SEM analysis:** Incorporation of TiO_2_ within the GIC matrix and revealed fewer surface cracks in modified specimens, indicating improved structural integrity. **Energy-dispersive spectroscopy analysis (EDS):** Confirmation of successful incorporation of TiO_2_-NT into the GIC matrix, with titanium content increasing proportionally to the amount of TiO_2_ added. **Raman spectroscopy:** The presence of TiO_2_-NT in the GIC showed that increasing TiO_2_ concentrations altered the chemical structure of the cement matrix. **Water sorption:** TiO_2_-NT incorporation did not significantly affect the water sorption of the glass ionomer cement. **Water solubility:** TiO_2_-NT incorporation reduced the solubility of the glass ionomer cement, with the greatest reduction observed at the 5% TiO_2_-NT concentration.	n/a	**Optical properties:** TiO_2_-NT alter optical properties by reducing luminosity and modifying color parameters. The effects were concentration-dependent, with higher TiO_2_ nanoparticles levels producing more pronounced changes. **Radiopacity:** TiO_2_ addition increased radiopacity, particularly at intermediate concentrations. **Setting time:** TiO_2_ incorporation had a limited effect on the setting behavior of the GIC. While the final setting time remained unchanged across all groups, the highest TiO_2_ concentration significantly prolonged the initial setting time.
Hepdeniz et al. (2021) [92]	Ionofil U (Voco, Cuxhaven, Germany)	3 wt% TiO_2_	**Surface hardness:** Slight, statistically insignificant increase compared to control	**SEM analysis:** SEM confirmed uniform, granular TiO_2_ distribution with no particle clustering. Surface cracks were observed in the GIC groups, and were more pronounced in the TiO_2_-modified specimens	n/a	n/a
Panahandeh et al. (2024) [93]	Fuji II (GC Corporation, Tokyo, Japan)	3, 5, and 10 wt% TiO_2_	**Flexural strength:** 5% TiO_2_ significantly higher than control and 3% TiO_2_ groups; 3% TiO_2_ and 10% TiO_2_ groups showed no significant difference vs. control Highest at 1 week: 5% group (45.32 ± 7.54 MPa) **Surface hardness:** All TiO_2_ concentrations exhibited decreased hardness vs. control Lowest hardness: 10% TiO_2_ group at 1 week (16.12 ± 4.28 VHN)	n/a	n/a	n/a

## Data Availability

No new data were created or analyzed in this study. Data sharing is not applicable to this article.

## Supplementary Materials

The following supporting information can be downloaded at: https://www.mdpi.com/article/10.3390/ma19132827/s1, Table S1: PRISMA Checklist; Table S2: General characteristics of included studies; Table S3: Quality Assessment.

## Abbreviations

The following abbreviations are used in this manuscript:

GIC	Glass ionomer cement
TiO_2_	Titanium dioxide
TiO_2_-NT	Titanium dioxide nanotubes
TiO_2_-NPs	Titanium dioxide nanoparticles
TiO_2_-MPs	Titanium dioxide microparticles
ECM	Extracellular matrix
SEM	Scanning electron microscopy
EDS/EDX	Energy-dispersive X-ray spectroscopy
AFM	Atomic force microscopy
CFU	Colony-forming units
MSBS	Microshear bond strength
VEGF	Vascular endothelial growth factor
TNF-α	Tumor necrosis factor alpha
LPS	Lipopolysaccharide
