# Supplementary material for: Effect of Titanium Dioxide (TiO2) Incorporation on the Properties of Glass Ionomer Cements: A Systematic Review
Source: Materials (Basel). 2026 Jul 2;19(13):2827. doi: 10.3390/ma19132827 (PMC13362690; doi:10.3390/ma19132827)
Supplement: Supplementary file 1 [file materials-19-02827-s001.zip › materials-4372435-supplementary.pdf]

Table S1. PRISMA Checklist

Topic	No.	Item	Location where item is reported
**TITLE**			
**Title**	1	Identify the report as a systematic review.	1
**ABSTRACT**			
**Abstract**	2	See the PRISMA 2020 for Abstracts checklist	
**INTRODUCTION**			
**Rationale**	3	Describe the rationale for the review in the context of existing knowledge.	1-3
**Objectives**	4	Provide an explicit statement of the objective(s) or question(s) the review addresses.	3
**METHODS**			
**Eligibility criteria**	5	Specify the inclusion and exclusion criteria for the review and how studies were grouped for the syntheses.	4-5
**Information sources**	6	Specify all databases, registers, websites, organisations, reference lists and other sources searched or consulted to identify studies. Specify the date when each source was last searched or consulted.	5
**Search strategy**	7	Present the full search strategies for all databases, registers and websites, including any filters and limits used.	5

Topic	No.	Item	Location where item is reported
**Selection process**	8	Specify the methods used to decide whether a study met the inclusion criteria of the review, including how many reviewers screened each record and each report retrieved, whether they worked independently, and if applicable, details of automation tools used in the process.	N/A
**Data collection process**	9	Specify the methods used to collect data from reports, including how many reviewers collected data from each report, whether they worked independently, any processes for obtaining or confirming data from study investigators, and if applicable, details of automation tools used in the process.	5
**Data items**	10a	List and define all outcomes for which data were sought. Specify whether all results that were compatible with each outcome domain in each study were sought (e.g. for all measures, time points, analyses), and if not, the methods used to decide which results to collect.	N/A
	10b	List and define all other variables for which data were sought (e.g. participant and intervention characteristics, funding sources). Describe any assumptions made about any missing or unclear information.	N/A
**Study risk of bias assessment**	11	Specify the methods used to assess risk of bias in the included studies, including details of the tool(s) used, how many reviewers assessed each study and whether they worked independently, and if applicable, details of automation tools used in the process.	5-6
**Effect measures**	12	Specify for each outcome the effect measure(s) (e.g. risk ratio, mean difference) used in the synthesis or presentation of results.	N/A
**Synthesis methods**	13a	Describe the processes used to decide which studies were eligible for each synthesis (e.g. tabulating the study intervention characteristics and comparing against the planned groups for each synthesis (item 5)).	5

Topic	No.	Item	Location where item is reported
	13b	Describe any methods required to prepare the data for presentation or synthesis, such as handling of missing summary statistics, or data conversions.	5
	13c	Describe any methods used to tabulate or visually display results of individual studies and syntheses.	5
	13d	Describe any methods used to synthesize results and provide a rationale for the choice(s). If meta-analysis was performed, describe the model(s), method(s) to identify the presence and extent of statistical heterogeneity, and software package(s) used.	N/A
	13e	Describe any methods used to explore possible causes of heterogeneity among study results (e.g. subgroup analysis, meta-regression).	N/A
	13f	Describe any sensitivity analyses conducted to assess robustness of the synthesized results.	N/A
**Reporting bias assessment**	14	Describe any methods used to assess risk of bias due to missing results in a synthesis (arising from reporting biases).	6
**Certainty assessment**	15	Describe any methods used to assess certainty (or confidence) in the body of evidence for an outcome.	N/A
**RESULTS**			
**Study selection**	16a	Describe the results of the search and selection process, from the number of records identified in the search to the number of studies included in the review, ideally using a flow diagram.	6
	16b	Cite studies that might appear to meet the inclusion criteria, but which were excluded, and explain why they were excluded.	N/A
**Study characteristics**	17	Cite each included study and present its characteristics.	6-10

Topic	No.	Item	Location where item is reported
**Risk of bias in studies**	18	Present assessments of risk of bias for each included study.	10
**Results of individual studies**	19	For all outcomes, present, for each study: (a) summary statistics for each group (where appropriate) and (b) an effect estimate and its precision (e.g. confidence/credible interval), ideally using structured tables or plots.	6-25
**Results of syntheses**	20a	For each synthesis, briefly summarise the characteristics and risk of bias among contributing studies.	N/A
	20b	Present results of all statistical syntheses conducted. If meta-analysis was done, present for each the summary estimate and its precision (e.g. confidence/credible interval) and measures of statistical heterogeneity. If comparing groups, describe the direction of the effect.	N/A
	20c	Present results of all investigations of possible causes of heterogeneity among study results.	N/A
	20d	Present results of all sensitivity analyses conducted to assess the robustness of the synthesized results.	N/A
**Reporting biases**	21	Present assessments of risk of bias due to missing results (arising from reporting biases) for each synthesis assessed.	10
**Certainty of evidence**	22	Present assessments of certainty (or confidence) in the body of evidence for each outcome assessed.	N/A
**DISCUSSION**			
**Discussion**	23a	Provide a general interpretation of the results in the context of other evidence.	26-28
	23b	Discuss any limitations of the evidence included in the review.	28
	23c	Discuss any limitations of the review processes used.	28

Topic	No.	Item	Location where item is reported
	23d	Discuss implications of the results for practice, policy, and future research.	28
**OTHER INFORMATION**			
**Registration and protocol**	24a	Provide registration information for the review, including register name and registration number, or state that the review was not registered.	N/A
	24b	Indicate where the review protocol can be accessed, or state that a protocol was not prepared.	N/A
	24c	Describe and explain any amendments to information provided at registration or in the protocol.	N/A
**Support**	25	Describe sources of financial or non-financial support for the review, and the role of the funders or sponsors in the review.	N/A
**Competing interests**	26	Declare any competing interests of review authors.	N/A
**Availability of data, code and other materials**	27	Report which of the following are publicly available and where they can be found: template data collection forms; data extracted from included studies; data used for all analyses; analytic code; any other materials used in the review.	N/A

Topic	No.	Item	Reported?
**TITLE**			
**Title**	1	Identify the report as a systematic review.	Yes
**BACKGROUND**			
**Objectives**	2	Provide an explicit statement of the main objective(s) or question(s) the review addresses.	Yes

Topic	No.	Item	Reported?
**METHODS**			
**Eligibility criteria**	3	Specify the inclusion and exclusion criteria for the review.	No
**Information sources**	4	Specify the information sources (e.g. databases, registers) used to identify studies and the date when each was last searched.	Yes
**Risk of bias**	5	Specify the methods used to assess risk of bias in the included studies.	Yes
**Synthesis of results**	6	Specify the methods used to present and synthesize results.	Yes
**RESULTS**			
**Included studies**	7	Give the total number of included studies and participants and summarise relevant characteristics of studies.	Yes
**Synthesis of results**	8	Present results for main outcomes, preferably indicating the number of included studies and participants for each. If meta-analysis was done, report the summary estimate and confidence/credible interval. If comparing groups, indicate the direction of the effect (i.e. which group is favoured).	Yes
**DISCUSSION**			
**Limitations of evidence**	9	Provide a brief summary of the limitations of the evidence included in the review (e.g. study risk of bias, inconsistency and imprecision).	No
**Interpretation**	10	Provide a general interpretation of the results and important implications.	Yes
**OTHER**			
**Funding**	11	Specify the primary source of funding for the review.	No
**Registration**	12	Provide the register name and registration number.	No

Table S2. General characteristics of included studies.

Study	Aim of the study	Material and Methods	Results	Conclusions
Assery et al. (2020) [61]	To evaluate how adding silver and titanium dioxide nanoparticles to conventional glass ionomer cement (GIC) affects its mechanical properties.	Experimental groups were made by adding 5 wt.% Ag or TiO_2 nanoparticles to GIC powder. The storage of the specimens was in water at 37 °C for 24 and tested using mechanical and microhardness methods.	Adding Ag or TiO_2 nanoparticles improved compressive strength, tensile strength, and hardness of GIC, with TiO_2 showing the greatest overall improvement, while flexural strength showed no significant change.	Reinforcing GIC with Ag or TiO_2 nanoparticles improved compressive strength, tensile strength, and hardness, but not flexural strength, suggesting their potential use in high stress-bearing clinical applications.
Cibim et al. (2017) [48]	To evaluate the physicochemical and biological properties of conventional glass ionomer cement (GIC) modified with varying concentrations of TiO_2 nanotubes.	A conventional glass ionomer cement was modified with 3%, 5%, and 7% TiO_2 nanotubes. The physical and chemical properties were evaluated using energy-dispersive spectroscopy, surface roughness, surface hardness, and fluoride release tests. Biological performance was assessed for 3% and 5% TiO_2 groups through cytotoxicity as well as cell morphology, and extracellular matrix (ECM) formation using human gingival fibroblasts.	Incorporating TiO_2 nanotubes into GIC did not change surface topography or roughness. Hardness was highest in the 5% TiO_2 group. Fluoride release increased in all TiO_2-containing groups compared to control, especially during the first 48 hours. The 5% TiO_2 group showed the highest cell viability, and TiO_2 did not affect cell adhesion or morphology. Extracellular matrix analysis showed that GIC, particularly with 5% TiO_2, promoted collagen-rich ECM over time.	Adding 5% TiO_2 nanotubes to conventional GIC enhances microhardness, fluoride release, and ECM composition without affecting surface roughness, indicating its suitability for high stress-bearing restorations.
Cvjeticanin et al. (2024) [62]	To assess the effects of TiO_2 and Mg-doped hydroxyapatite (NPs) nanoparticles on cell viability and ion release in two conventional glass ionomer cements.	Two conventional GICs were modified with 5 wt% of TiO_2 and Mg-doped hydroxyapatite nanoparticles and characterized by Raman spectroscopy, X-ray diffraction, and SEM. Biocompatibility of modified GIC was evaluated using ATP-based assays on human dental pulp stem cells, and ion release (Ca, P, Al, Mg, Ti, F) was measured over 14	Biocompatibility tests revealed that all Ketac-modified groups reduced cell viability more than Fuji groups, with some samples dropping below 50%. Ion release differed between GICs: Ketac released more Ca, Mg, and Al, while Fuji released more Ti and fluoride initially. Fluoride release peaked at 24 hours in all groups, then decreased over 14 days.	Incorporating TiO_2 and Mg-doped HAp nanoparticles into Fuji IX and Ketac Molar GICs did not compromise biocompatibility or fluoride release, though cell viability varied between cements, and all maintained their anti-cariogenic potential.

		days using ICP-OES and ion chromatography.		
Fathi et al. (2022) [63]	To investigate the effect of incorporating 3 wt% and 5 wt% TiO_2 nanoparticles on the mechanical and physical properties of glass ionomer cement.	Titanium dioxide nanoparticles were added to conventional GIC. Samples were molded, stored in water at 37 °C, and tested for surface microhardness, water sorption/solubility, and flexural strength using standard Vickers and three-point bending methods.	Incorporating 3% and 5% TiO_2 nanoparticles into GIC significantly increased surface microhardness. Flexural strength showed no significant changes among the groups. Water sorption and solubility were significantly reduced in the modified GICs compared to the control, indicating improved resistance to moisture uptake and dissolution.	GIC modified with 3% and 5% (w/w) TiO_2 nanoparticles shows enhanced mechanical properties and can be regarded as a suitable option for restorative applications.
Gjorgievska et al. (2020) [64]	To investigate how incorporation of Al_2O_3, ZrO_2 and TiO_2 nanoparticles affect the properties of conventional glass ionomer cements.	Two conventional glass ionomer cements were investigated in control and nanoparticle-modified forms (Al_2O_3, ZrO_2, TiO_2 at 2, 5, and 10 wt%). Compressive strength was evaluated after 1 day and 1 week. Microstructural features of fractured surfaces were examined by scanning electron microscopy, and elemental ion release into saline after 1 week was measured using inductively coupled plasma analysis.	The incorporation of Al_2O_3, ZrO_2, and TiO_2 nanoparticles influenced both the microstructure and performance of glass ionomer cements. SEM observations indicated a more compact cement matrix with reduced porosity and fewer microcracks. Compressive strength improved in cements containing ZrO_2 and was highest with TiO_2, whereas Al_2O_3 slightly diminished strength. Overall ion release was minimal, with only a modest increase in aluminium ions from Al_2O_3-modified cements, while zirconium and titanium ions were largely undetectable.	Adding Al_2O_3, ZrO_2, and TiO_2 nanoparticles to high-viscosity GICs reduces internal porosity, can boost compressive strength, and enhances overall material properties without detectable ion release, suggesting improved suitability for clinical use.
Garcia-Contreras et al. [86] (2015)	To assess the effect of 3 wt% and 5 wt%TiO_2 nanoparticles on the physical properties, antibacterial activity, and bond strength of conventional glass ionomer cements.	Three types of conventional GIC (core shade base cement-grey, base cement- yellow and FX-II Enhanced restoration (A2) powders were modified with 3% and 5% TiO_2 nanoparticles. Specimens were	Adding TiO_2 nanoparticles had no effect on the mechanical, antibacterial, or bond properties of base/liner and core build GICs. However, for restorative GIC, TiO_2 significantly enhanced	TiO_2 nanoparticle–modified GIC demonstrates enhanced mechanical durability and antibacterial effects, indicating its potential as a reliable restorative

		prepared for Vickers microhardness, compressive, and flexural strength tests. Shear bond strength was tested on bovine enamel and dentin, while antibacterial activity against *Streptococcus mutans* was evaluated via agar diffusion. SEM and EDS analyses examined surface morphology and elemental composition.	microhardness, flexural and compressive strength, and antibacterial activity, while maintaining normal adhesion to enamel and dentin.	material for long-lasting, functional dental restorations.
Gjorgievska et al. [87] (2015)	To investigate the influence of incorporation of 10 wt% Al_2O_3, ZrO_2 or TiO_2 nanoparticles on the properties of conventional glass ionomer cements.	Two commercial GICs were tested as controls and after modification with 10 wt% Al_2O_3, ZrO_2, or TiO_2 nanoparticles. Cylindrical specimens were prepared, stored in saline for one week, and tested for compressive strength. Nanoparticles and fractured samples were analyzed using SEM, and energy-dispersive X-ray analysis (EDX).	Compressive strength increased with TiO_2 addition in both GICs and with ZrO_2 in EQUIA Fil, while Al_2O_3 had no significant effect. SEM revealed reduced air voids and fewer, smaller cracks in nanoparticle-modified cements. EDX confirmed typical GIC elements, with Zr or Ti detected in modified samples and no evidence of chemical interaction with the cement matrix.	Nanoparticle modification improved GIC microstructure and, in some cases, strength, without chemical interaction with the cement matrix.
Hamid et al. (2019) [65]	To assess how adding titanium dioxide nanoparticles and cetylpyridinium chloride (CPC) affects the strength and antibacterial properties of conventional glass-ionomer cement.	Conventional GIC was tested alone and after modification with 3 wt% TiO_2 nanoparticles or 1 wt% CPC. Compressive strength was measured on cylindrical specimens using a universal testing machine, and antibacterial activity against *Streptococcus mutans* was evaluated by the agar diffusion method.	Adding 3 wt% TiO_2 nanoparticles significantly improved the compressive strength of GIC, while CPC had no effect on strength. TiO_2-modified GIC also showed significantly higher antibacterial activity than conventional GIC. CPC slightly enhanced antibacterial activity, but the increase was not statistically significant.	Adding 3% TiO_2 nanoparticles enhanced both the strength and antibacterial activity of GIC.
Hussein et al. (2022) [66]	To evaluate water sorption and water solubility of conventional	Sixty GIC discs were prepared and divided into two groups: conventional (control) and TiO_2	TiO_2-modified GIC showed lower water sorption and solubility than conventional GIC, especially in	TiO_2 nanoparticles reduce water sorption and solubility in GIC, with the highest absorption in the

	glass ionomer cement modified with TiO_2 nanoparticles.	nanoparticle-modified GIC. Each group was further split into three subgroups (n=10) based on a storage solution: artificial saliva, alcohol-containing mouthwash, or alcohol-free mouthwash. Water sorption and solubility were measured after 24 hours, 1 week, and 1 month of immersion.	artificial saliva and alcohol-containing solutions. Both materials absorbed less water in artificial saliva, and TiO_2 GIC also showed reduced water uptake in alcohol-free mouthwash. Over time, solubility gradually increased for conventional GIC in saliva and TiO_2 GIC in alcohol.	first 24 h and stabilization of modified GIC after 1 month.
Ibrahim et al. (2017) [67]	To evaluate the antibacterial activity against *S. mutans* biofilms and the mechanical properties of a novel GIC dually modified with chitosan (CH) and TiO_2 nanoparticles.	Conventional GIC was modified with 3% (w/w) TiO_2 nanoparticles, 10% (v/v) CH solutions, or dual TiO_2/CH modification, with the unmodified material serving as a control. Biofilm formation was assessed using SEM, a live/dead assay with confocal microscopy, colony-forming unit counts, and an (MTS assay. Bulk and surface mechanical properties were evaluated through flexural strength, compressive strength, and surface hardness testing.	Modification of the GIC with TiO_2 reduced bacterial biofilm formation compared with the non-modified GIC, as demonstrated by confocal microscopy and SEM. However, no significant differences were observed between the TiO_2-modified and non-modified GIC in CFU counts or MTS assays. In addition, TiO_2 modification improved flexural and compressive strength without significantly affecting surface hardness compared with the non-modified GIC.	Dual modification of GIC with TiO_2 and CH enhanced antibacterial activity against *S. mutans* biofilms and improved mechanical properties. TiO_2 mainly strengthened mechanical performance, while CH predominantly boosted antibacterial effects.
Ivanišević et al. (2021) [68]	To evaluate the compressive strength (CS), breaking strength (BS), and compressive modulus (CM) of conventional glass ionomer cement (GIC) modified with TiO_2 nanoparticles, marine-derived hydroxyapatite (md-HAp) microparticles, and their combined incorporation.	Four groups of GIC were prepared: a control group without particle addition (commercially available conventional GIC) and three experimental groups in which the powder was modified with 3 wt% commercially available TiO_2, 3 wt% HAp synthesized from cuttlefish bone, or a combination of 1.5 wt% TiO_2 and 1.5 wt% HAp. For CS, BS, and CM testing, cylindrical specimens were fabricated for each group. All	The unmodified GIC control group demonstrated the highest compressive strength, breaking strength, and compressive modulus. Tukey's post hoc analysis revealed statistically significant differences between the control group and all experimental groups for all tested mechanical parameters, whereas no statistically significant differences were observed among the experimental groups.	The addition of TiO_2 nanoparticles and marine-derived hydroxyapatite, either individually or in combination, did not improve the mechanical properties of GIC.

		measurements were performed in accordance with ISO specifications. The data were statistically analyzed using descriptive statistics.		
Kantovitz et al. (2020) [69]	To evaluate the effect of incorporating titanium dioxide nanotubes (TiO_2-nt) into conventional high-viscosity glass ionomer cement (GIC) on its physico-mechanical properties, including compressive strength (CS), flexural strength (FS), microshear bond strength and failure mode to dentin (MSBS), as well as surface roughness and weight loss before and after brushing simulation.	TiO_2 nanotubes were incorporated into Ketac Molar GIC, generating four groups: control and TiO_2-nt–modified (3%, 5%, 7%). CS, FS, MSBS, and surface roughness and weight loss before/after brushing simulation were evaluated per group. Mechanical tests were conducted according to ISO standards using a universal testing machine. For bond testing, human third molars were restored on medium dentin surfaces. Failure mode was assessed stereomicroscopically and validated by SEM. Brushing simulation simulated three years of clinical brushing. Structural effects of TiO_2-nt incorporation were analyzed by SEM.	The 5% TiO_2-nt group exhibited the highest compressive strength. Microshear bond strength was higher for 5% TiO_2-nt compared with 7%, with no differences versus 3% or control. TiO_2-nt significantly reduced matrix weight loss irrespective of concentration, with the lowest loss at 5%. SEM analysis revealed no substantial structural alterations following TiO_2-nt incorporation.	TiO_2-nt incorporation improved compressive strength and reduced brushing-induced weight loss without affecting adhesion, flexural strength, roughness, or structure, supporting its potential to enhance GIC clinical performance.
Laiteerapong et al. (2018) [70]	To evaluate the genotoxic effects of conventional glass ionomer cement (GIC) and GIC modified with titanium dioxide nanoparticles (TiO_2NPs) or microparticles (TiO_2MPs) on human gingival fibroblasts (HGFs), and to compare the genotoxic differences between the tested materials.	TiO_2NPsGIC and TiO_2MPsGIC were formulated by incorporating 10% (w/w) TiO_2NPs or TiO_2MPs. Dulbecco's Modified Eagle Medium (DMEM) served as the culture medium for HGFs and was also used for eluate preparation. Eluates obtained from all experimental groups were subjected to the XTT cell viability assay to determine EC50 values. DNA double-strand breaks (DSBs)	Eluate concentrations of 20% and 5% were selected for the γ-H2AX immunofluorescence assay. At the 20% concentration, exposure to eluates from both TiO_2NPsGIC and TiO_2MPsGIC resulted in a significantly lower mean number of foci per cell and a higher proportion of foci-free cells compared with the conventional GIC and culture medium groups.	Neither conventional GIC nor 10% TiO2-modified GICs induced genotoxic effects in human gingival fibroblasts. Both TiO_2NPsGIC and TiO_2MPsGIC showed lower genotoxicity than unmodified GIC, with no significant difference between nanoparticle- and microparticle-containing formulations.

		in HGFs were subsequently evaluated using the γ-H2AX immunofluorescence assay. Cells cultured on microscope slides were examined with a fluorescence imaging microscope, and γ-H2AX foci were quantitatively evaluated by direct visual counting from the acquired images.		
Mahendra et al. (2023) [71]	To evaluate titanium ion release into artificial saliva from glass ionomer cement (GIC) modified with 3% and 5% (w/w) TiO_2 nanoparticles (TiO_2-NPs) and to assess its antibacterial activity against *S. mutans*.	A total of 120 cylindrical specimens (4 mm diameter, 6 mm height) were fabricated from GIC modified with 3% and 5% (w/w) TiO_2-NPs and allocated into two groups (n = 60). Samples were immersed in synthetic saliva, and titanium ion release was quantified at 24 hours, 2, 4, and 6 months using inductively coupled plasma mass spectrometry (ICP-MS). Antibacterial activity was assessed by colony-forming unit (CFU) analysis.	Titanium ion release was highest within the first two months, peaking at month two, and was consistently greater in the 5% TiO_2 group than in the 3% group. The 3% group showed no significant release at later intervals, whereas the 5% group demonstrated significant release at all time points. Increased TiO_2 concentration (3% to 5%) was associated with enhanced antibacterial activity against *S. mutans*, although CFU counts decreased initially and then gradually increased over time.	Titanium ion release peaked at two months and was consistently higher in 5% than 3% TiO_2-NP–modified GIC; both 3% and 5% (w/w) formulations were safe and demonstrated significant antimicrobial activity.
Mansoor et al. (2024) [72]	To investigate the synthesis and characterization of biocompatible TiO_2 nanoparticles (TiO_2-NPs) using the probiotic *Bacillus coagulans*, to evaluate their incorporation into conventional glass ionomer cement (GIC), and to assess the effects of varying TiO_2 nanoparticle concentrations on cell viability, microhardness, and surface morphology.	TiO_2-NPs were biosynthesized using *Bacillus coagulans*. Their crystalline structure and size were characterized by X-ray diffraction (XRD) and dynamic light scattering (DLS), while morphology was analyzed using scanning electron microscopy (SEM) and atomic force microscopy (AFM). Chemical composition and purity were assessed by Fourier-transform infrared spectroscopy (FTIR),	The results demonstrated that the synthesized TiO_2 nanoparticles formed smooth, spherical agglomerates and clusters. The nanoparticles exhibited over 90% cell viability, confirming their non-cytotoxic nature. Among the tested groups, glass ionomer cement reinforced with 5% TiO_2 nanoparticles showed the highest microhardness compared to the control and other experimental groups. Surface morphology	The incorporation of biologically synthesized TiO_2-NPs into GIC enhanced its structural integrity and mechanical performance while maintaining biological compatibility. These findings support the potential application of bio-derived TiO_2 nanoparticles as effective and reliable additives in dental restorative materials.

		energy-dispersive X-ray spectroscopy (EDX), and UV–Vis diffuse reflectance spectroscopy (DRS). Cytotoxicity was evaluated on L929 mouse fibroblasts using the MTT assay. For mechanical testing, 50 glass ionomer cement (GIC) samples containing 0%, 3%, 5%, 7%, and 10% (w/w) TiO_2-NPs were prepared and assessed for microhardness using the Vickers test and SEM analysis.	analysis further revealed a reduction in cracks.	
Mansoor et al. (2022) [73]	To assess the physicochemical properties of a novel glass ionomer cement (GIC) incorporating TiO_2 nanoparticles (TiO_2-NPs) biosynthesized by *Bacillus subtilis*, by evaluating the mechanical strength, surface morphology, biocompatibility, shelf life, and durability of the modified GIC as an improved dental restorative material.	TiO_2-NPs were synthesized by inoculating a fresh culture of *Bacillus subtilis* into nutrient broth and incubating it for 24 hours. The obtained TiO_2-NPs were incorporated into a glass ionomer cement (GIC) restorative material to prepare 100 specimens with varying nanoparticle concentrations (0, 3, 5, 7, and 10 wt% TiO_2-GIC). The mechanical properties of the modified GIC samples were then evaluated using a universal testing machine.	Among the tested formulations, 5 wt% TiO_2-GIC showed the most favorable performance for dental caries treatment without detectable cytotoxicity. Mechanical testing revealed that compressive strength increased with TiO_2 content up to 5 wt%, followed by a decline at higher concentrations, while flexural strength improved progressively in samples containing 0%, 3%, and 5 wt% TiO_2.	*Bacillus subtilis*-derived TiO_2 nanoparticles exhibit strong potential for the development of next-generation GIC restorative materials with enhanced mechanical performance and biocompatibility for dental applications.
Meyer et al. (2025) [74]	To evaluate the effect of incorporating titanium dioxide nanotubes (TiO_2-nt) into a high-viscosity glass ionomer cement (GIC) on the biological response and inflammatory marker expression of pre-odontoblastic cells, with and without LPS stimulation.	GIC modified with 3%, 5%, or 7% TiO_2-nt, were investigated in the presence or absence of *Bacteroides forsythus* LPS. Experimental groups included GIC alone (control), GIC+LPS, and each TiO_2-nt concentration with and without LPS; MDPC-23 cells without GIC served as blank control. Assessed outcomes comprised proliferation/viability (trypan blue)	Cell proliferation increased over time in all groups and was not affected by TiO_2-nt incorporation. GIC samples showed reduced metabolic activity (MTT) compared with cells cultured without GIC discs, irrespective of TiO_2-nt presence. However, TiO_2-nt attenuated the GIC-induced increase in selected immune-inflammatory biomarkers. LPS	Incorporating TiO_2-nt into GIC did not affect cell proliferation, viability, metabolism, or morphology, but showed potential to modulate immune-inflammatory and odontoblastic differentiation markers.

		and metabolism (MTT), morphology by confocal microscopy, protein levels (IL-1β, IL-6, IL-10, VEGF, TNF-α) via multiplex assay, and gene expression (RT-PCR) of inflammatory markers.	exposure significantly altered inflammatory marker expression. No morphological differences were observed among groups.	
Morales-Valenzuela et al. (2022) [75]	To evaluate the fluoride release, fluoride recharge capacity, and cytotoxicity of glass ionomer cements (GIC) reinforced with TiO_2 nanoparticles (TiO_2N).	Four conventional GICs were tested either in their original form (control groups) or after incorporation of TiO_2N (experimental groups). Disc-shaped specimens (5 mm in diameter, 1 mm thick; n = 10 per subgroup) were prepared, yielding a total of 80 samples (40 controls and 40 TiO_2N–modified materials). Fluoride release was measured at 1, 2, 6, 10, 31, 90, 180, 240, and 300 days. Fluoride recharge was performed on days 30 and 179 by immersing specimens in 1 mL of 20,000 ppm sodium fluoride gel. Cytotoxicity was assessed using gingival fibroblasts and quantified with the MTT cell viability assay.	TiO_2N–modified groups demonstrated higher and more stable fluoride release compared with controls. Following the first recharge, three out of four experimental groups exhibited significantly enhanced recharge capacity, while the second recharge yielded superior performance in only one group. No cytotoxic effects were detected in all experimental groups, although two groups showed significant differences compared with controls.	Incorporation of TiO_2N into GIC significantly enhanced fluoride release and recharge capacity without inducing cytotoxic effects, suggesting improved anti-demineralization potential and maintained biocompatibility.
Ramić et al. (2024) [76]	To compare the effects of adding 5 wt% TiO_2, MgHAp100, or MgHAp1000 nanoparticles on the fracture toughness, flexural strength, Vickers microhardness, and rheological properties of two commercially available high-viscosity GICs (Fuji IX and Ketac Molar) relative to their unmodified controls.	Two glass ionomer cements, Fuji IX and Ketac Molar, were modified by replacing 5 wt% of the powder with TiO_2, MgHAp100, or MgHAp1000 nanoparticles, yielding eight groups including unmodified controls. Nanoparticles were blended into the powder, mixed with the corresponding liquid according to the manufacturers' powder-to-	TiO_2 addition increased fracture toughness in Ketac Molar and microhardness in Fuji IX ($p < 0.05$) but did not affect flexural strength in either material. MgHAp100 reduced flexural strength, increased microhardness in Fuji IX, but decreased microhardness in Ketac Molar. MgHAp1000 showed no effects on any mechanical property. MgHAp1000 produced the greatest	Modification of nanoparticles showed inconsistent results depending on the material. No type of nanoparticles uniformly improved all mechanical properties of both composite cements, which emphasizes that predictable bonding and uniform distribution of nanoparticles in the cement matrix are essential.

		liquid ratios, and specimens were stored at 37°C for 24 h. Mechanical properties (flexural strength, fracture toughness, Vickers microhardness), rheological behavior, and microstructure were evaluated using SEM and XRD.	increase in viscosity and storage modulus across both GICs. SEM confirmed TiO_2 agglomerate formation with interfacial cracks, while MgHAp particles showed better matrix adhesion but left unreacted glass cores visible in Ketac Molar.	
Rangel-Coelho et al. (2024) [77]	To assess whether adding TiO_2 nanotubes at increasing concentrations (3%, 5%, 7% wt) to a GIC would affect fibroblast behavior cell growth, mitochondrial function, morphology, and inflammatory cytokine activity both with and without LPS stimulation.	TiO_2 particles were incorporated into Ketac Molar powder at 3%, 5%, and 7% wt by vortex mixing, generating eight groups (with and without LPS). Specimens were prepared at a 1:1 powder/liquid ratio and stored at 37°C for 24 h. NIH/3T3fibroblasts were cultured directly on the discs and exposed to LPS (2 μg/mL) for 24 h. Evaluations included cell proliferation and mitochondrial activity, cell morphology, cytokine secretion (IL-1β, IL-6, IL-10, VEGF, TNF-α), and corresponding gene expression.	Cell proliferation and mitochondrial activity increased over time in all groups, peaking at 72 h, unaffected by TiO_2-nt or LPS. No morphological changes were observed. In LPS-challenged cells, TiO_2-nt reduced TNF-α at all concentrations at 12 h, IL-6 at 3–5% (12 h) and 5–7% (18 h), and IL-1β at all concentrations at 18 h. Without LPS, TiO_2-nt increased IL-6 and VEGF secretion. Gene expression confirmed that 5% and 7% TiO_2-nt significantly lowered VEGF levels in LPS-challenged cells at 72–120 h.	TiO_2-nt did not negatively affect the viability or morphology of fibroblasts and exhibited a concentration- and time-dependent ability to reverse LPS-induced expression of proinflammatory cytokines, confirming its biological safety and potential immunomodulatory benefits when incorporated into GIC.
de Gois Sena et al. (2024) [78]	To assess how different concentrations of TiO_2 nanotubes ($nTiO_2$) added to a GIC affect the growth, viability, and morphology of *Lactobacillus acidophilus* during early biofilm formation.	Ketac Molar EasyMix was modified with 3%, 5%, or 7% n-TiO_2 and compared with an unmodified control. Nanoparticles were blended into the powder by vortex mixing, specimens were prepared according to the manufacturer's instructions, and stored at 37°C for 24 h. *Lactobacillus acidophilus* (1×10^8 CFU/mL) biofilms were evaluated after 1, 3, and 7 days using agar diffusion and Live/Dead viability assays and	No differences in inhibition zone were found between GIC and $nTiO_2$ groups at any time point. Cell viability decreased with increasing $nTiO_2$ concentration; 7% $nTiO_2$ significantly reduced total bacterial colonies on Day 1 and viable bacteria on Day 3. At Day 7, GIC+5% $nTiO_2$ showed the lowest viable count and was the only group with a declining CFU trend over time. Cell morphology unaffected.	While $nTiO_2$ did not improve GIC's inhibition zone, 5% $nTiO_2$ most effectively reduced *L. acidophilus* viability over time, potentially interfering with initial biofilm colonization. Combined with its known mechanical benefits, 5% $nTiO_2$ is the optimal concentration for GIC modification.

		SEM analysis of bacterial morphology.		
Showkat et al. (2023) [79]	To compare the flexural strength (FS) of conventional GIC modified with different combinations of TiO_2 nanopowder, nanohydroxyapatite (nHA), and chitosan (CH) added to the powder and/or liquid components.	72 specimens, 6 groups (n=12/group). Base: conventional GIC type IX (GC Corporation). Modifications: Group I control (unmodified); Group II powder + 3% TiO_2; Group III powder + 10% nHA; Group IV liquid + 10% CH; Group V powder + 3% TiO_2 + liquid + 10% CH; Group VI powder + 10% nHA + liquid + 10% CH. TiO_2: anatase, 10–20 nm; nHA: 20–80 nm; CH: 0.2 mg/mL in 0.3N acetic acid, diluted 1:10 in GIC liquid. FS measured by 3-point bending test.	All modified groups showed higher FS than the unmodified control (Group I: 5.26±1.03 MPa). Group V (3% TiO_2 + 10% CH) achieved the highest FS (29.42±3.35 MPa), followed by Group II (27.81±3.50 MPa), Group IV (24.33±4.23 MPa), Group VI (16.52±1.81 MPa), and Group III (10.97±1.96 MPa).	All modifications improved GIC flexural strength compared to the unmodified control. The dual modification of GIC powder with 3% TiO_2 and liquid with 10% CH yielded the greatest FS improvement, demonstrating a synergistic effect. nHA alone showed the least improvement among modified groups.
da Silva Morais et al. (2022) [80]	To investigate how addition of 5% TiO_2 nanotubes to GIC affects the surface composition of elements, initial working time, and aluminum release rate under simulated caries conditions.	Two groups: KM (Ketac Molar EasyMix) control and KM+5% TiO_2-nt. TiO_2-nts: 20 nm length, 10 nm diameter, alkaline synthesis; blended into GIC powder by vortex 2 min; powder:liquid ratio 1:1. Disc specimens (4 mm × 6 mm), petroleum jelly-coated, stored 37°C/24 h.	Initial working time was unaffected by TiO_2-nt (KM: 321.4±3.4 s vs KM+5%TiO_2-nt: 319.9±7.1 s). EDS showed comparable elemental composition between groups, with sodium as the only element affected at baseline. Al release in the demineralizing solution was significantly reduced in KM+5%TiO_2-nt at all time points: ~60% reduction on days 1–5 and 100% by day 7. No Al release was detected under remineralizing conditions in either group.	Adding 5% TiO_2-nt to GIC did not alter initial working time or overall elemental surface composition but significantly reduced aluminum release under acidic conditions.
de Souza Araujo et al. (2021) [81]	To investigate how different concentrations of TiO_2 incorporated into GIC affect *S. mutans* at cellular and molecular level.	4 groups: GIC control, GIC+3%, +5%, +7% n-TiO_2 (alkaline synthesis, ~20 nm; vortex-blended into powder 2 min; P/L 1:1). Base: Ketac Molar EasyMix. Disc	GIC+5% n-TiO_2 produced the largest inhibition zone; no time-dependent effect was observed. All n-TiO_2 groups reduced total cell counts, with GIC+3% and GIC+5%	GIC modified with 3% and 5% n-TiO_2 most effectively reduced *S. mutans* viability, altered cell morphology, and downregulated *covR* - a key virulence regulatory

		specimens stored 37°C/24 h, UV-exposed 15 min/side. Bacterial strain: *S. mutans* UA159 (1×10^8 CFU/mL), 37°C/10% CO_2. Assessments at days 1, 3 and 7: inhibition zone (agar diffusion, n=3), cell viability (Live/Dead fluorescence, n=5), morphology (SEM 2000×, n=3), and gene expression by RT-PCR) at 24 and 72 h	most effectively reducing viable bacteria at days 1 and 3. SEM showed morphological shift from spherical to rod-shaped organization at 3% and 5%, but not at 7%. *covR* was significantly downregulated by all concentrations at 24 h, with 3% additionally reducing *vicR* over time. *gtfB*, *gtfC*, and *gtfD* were unaffected.	gene - potentially interfering with biofilm formation.
Wassel et al. (2022) [82]	To evaluate how adding 5 wt% silver (Ag-NP) or titanium dioxide (TiO_2-NP) nanoparticles to conventional GIC affects its antibacterial activity against *S. mutans*, fluoride ion release, and compressive strength.	Three groups (n=30 each): Group C (unmodified GIC), Group Ag (GIC + 5 wt% Ag-NP, <100 nm), Group Ti (GIC + 5 wt% TiO_2-NP, <20 nm). Base: self-cure GIC Riva; NPs evenly blended into GIC powder; P/L ratio 2.17:1. Disc specimens (10×2 mm) and cylinders (6×4 mm), n=10/parameter. Assessments: antibacterial activity, fluoride release, compressive strength, after 7 days.	Both Ag and Ti groups showed larger inhibition zones than the control. Ag-NP increased fluoride release at all time points, while TiO_2-NP reduced it; however, cumulative 28-day fluoride release did not differ significantly between any group vs control. Compressive strength followed the order Ti>Ag>C; only Ti was higher than both Ag and control.	Adding 5 wt% TiO_2-NP or Ag-NP to conventional GIC significantly enhanced antibacterial activity against *S. mutans* and improved compressive strength without compromising overall fluoride release.
Karamüftüoğlu et al. (2026) [83]	To assess the influence of hemp-derived, green-synthesized TiO_2 nanoparticles on the properties of two commercially available glass ionomer cements intended for different clinical applications.	Two glass ionomer cements, Ketac Cem Radiopaque and Ketac Molar Easymix, were modified by incorporating 1%, 3%, and 5% (w/w) TiO_2 nanoparticles. Non modifies specimens served as controls. Surface roughness was measured using profilometry, flexural strength was assessed under three-point bending, and Vickers microhardness was determined. SEM and EDX analyses were performed to evaluate surface morphology and	1-3% w/w additions of TiO_2 generally improved flexural strength and microhardness and maintained improved surface roughness, whereas the highest loading (5%) tended to show diminished or more inconsistent mechanical performance. SEM and EDX analysis confirmed successful nanoparticle incorporation and showed changes in surface morphology consistent with particle reinforcement and altered fracture patterns compared to controls.	Hemp-derived TiO_2 nanoparticles improve GIC mechanical properties at low concentrations (1-3%), while higher loading reduces performance and increases surface roughness, making optimization essential.

		confirm elemental composition and successful nanoparticle incorporation.		
Abozaid et al. (2026) [84]	To exaluate if the addiotion of green-synthesized TiO_2 nanoparticles made from *Citrus aurantium* seed extract could improve the physical and mechanical properties of glass ionomer cement.	Green-synthesized TiO_2 nanoparticles were produced using *Citrus aurantium* seed extract and then incorporated into Fuji IX GP at 5% and 10% (w/w) to prepare control and experimental GIC groups. The modified cements were fabricated into standardized specimens and evaluated for flexural strength, microhardness, water sorption and solubility, and physicochemical properties using universal testing, hardness testing, and immersion-based measurements.	In Fuji IX GP, the 10% TiO_2 group showed the highest flexural strength, flexural modulus, and microhardness, while the 5% formulation generally showed intermediate performance between the control and 10% group. Both 5% and 10% TiO_2 significantly reduced water sorption and solubility compared with the control, with the 10% group showing the greatest improvement in stability.	Green-synthesized TiO_2 nanoparticles enhance the mechanical and stability properties of glass ionomer cements
Shubha et al. (2025) [85]	To investigate the influence of the addition of synthesized TiO_2 nanoparticles from plant extracts (*Piper betle* and *Ocimum sanctum*) into type-II glass ionomer cement and its influence on mechanical properties, setting time, antimicrobial activity, and antioxidant potential.	Biosynthesized TiO_2 nanoparticles were incorporated into the powder of GC Fuji II at different concentrations: F1: control; F2: 50 mg/g of hydrothermal TiO_2 NPs+GIC; F3: 100 mg/g hydrothermal TiO_2 NPs+GIC; F4: 50 mg/g of microwave TiO_2 NPs+GIC; F5: 100 mg/g of microwaved TiO_2 NPs+GIC, These were then evaluated in vitro for setting time,	TiO_2 nanoparticles incorporated into Fuji II significantly reduced setting time and increased compressive strength only at higher loading (100 mg/g), while lower concentrations showed no significant mechanical improvement. Antimicrobial testing showed marginal but statistically significant reductions in *S. mutans* and *L. acidophilus* at higher nanoparticle content, with minimal	Plant-synthesized TiO_2 nanoparticles can improve the setting behavior and compressive strength of type-II glass ionomer cement while offering only modest antimicrobial and antioxidant benefits, suggesting potential for dental use.

		compressive strength, antimicrobial activity against *Streptococcus mutans* and *Lactobacillus acidophilus* and antioxidant capacity using the DPPH radical scavenging assay.	overall enhancement at 50 mg/g. Antioxidant activity was low across all groups, with only slight increases in TiO_2-modified formulations compared to the control.	
Ganesh et al. (2026) [88]	To evaluate and compare the antimicrobial efficacy, mechanical properties, and surface characteristics of green-mediated titanium-modified glass ionomer cement (TiO_2-GIC) and green-mediated chitosan-modified glass ionomer cement (Chi-GIC).	Three groups were prepared: TiO_2-GIC, Chi-GIC (both 5 wt%), and conventional GIC (control). Material characterization was performed using FTIR, SEM, and EDS. Antibacterial activity against *Streptococcus mutans* and *Lactobacillus acidophilus* was assessed by agar well diffusion, while mechanical properties were evaluated through Vickers microhardness and compressive strength testing.	TiO_2-GIC demonstrated moderate antibacterial activity against *Streptococcus mutans* and *Lactobacillus acidophilus* and showed the best overall mechanical performance among the tested materials, exhibiting the highest microhardness and compressive strength along with the lowest surface roughness.	Results indicate that TiO_2 nanoparticle modification enhances the structural performance of GIC and supports its potential clinical use in improving restorative material durability.
Garcia-Contares et al. (2014) [89]	To investigate the potential cytotoxic and pro-inflammatory effects of three types of powdered glass ionomer cements (GIC) - base, core build, and restorative, prepared with and without titanium dioxide nanoparticles (TiO_2NPs).	Three types of GIC were modified with TiO_2NPs (anatase phase, <25 nm) at 3% and 5% (w/w). Cement blocks were prepared, ground into powder, and sterilized by autoclaving. Human oral squamous cell carcinoma cell lines (HSC-2, HSC-3, HSC-4, Ca9-22) and normal oral cells (HGF, HPC, HPLF) were exposed to different concentrations of the modified and unmodified cements. Cell viability was evaluated using the MTT assay, PGE2 levels by ELISA, and ultrastructural changes by transmission electron microscopy.	TiO_2NP incorporation into GIC did not significantly affect cytotoxic selectivity, as cancer cells remained more sensitive than normal oral cells, except for HGF exposed to FX-II with 5% TiO_2. TiO_2-modified FX-II maintained or enhanced IL1β-induced PGE_2 production, indicating synergistic pro-inflammatory effects. Ultrastructural analysis showed no TiO_2-dependent morphological differences, with both modified and unmodified FX-II causing similar dose-dependent cellular changes.	GICs modified with TiO_2 nanoparticles exhibit acceptable to moderate biocompatibility and may induce pro-inflammatory responses at higher concentrations.

Shahpaska et al. (2026) [90]	To assess the influence of TiO2 addition to conventional glass ionomer cements on their surface topography and microhardness.	Two conventional glass-ionomer cements, Ketac Universal and GC Fuji TRIAGE, were modified with Al_2O_3, ZrO_2, or TiO_2 nanoparticles at concentrations of 2, 5, and 10 wt%. A total of 120 disc-shaped specimens were prepared and stored in saline at 37°C. Vickers microhardness and surface topography (AFM) were evaluated on the same specimens after 1 and 21 days.	In GC Fuji TRIAGE, most nanoparticle additions generally increased microhardness, particularly at lower concentrations, while the effects after 21 days depended on the nanoparticle type and concentration. In Ketac Universal, nanoparticle incorporation generally reduced or did not significantly change microhardness, especially at higher concentrations. AFM analysis showed that nanoparticles altered surface morphology in both materials.	The effect of nanoparticle incorporation on glass-ionomer cements is material-, concentration-, and time-dependent, with potential benefits but no consistent improvement in surface hardness or roughness.
Kantovitz et al. (2023) [91]	To evaluate the effect of TiO_2 nanotube incorporation on the morphology, setting reaction, optical properties, water sorption, solubility, and chemical characteristics of a high-viscosity conventional glass-ionomer cement.	A high-viscosity conventional glass-ionomer cement, Ketac Molar EasyMix, was modified with TiO_2 nanotubes at 3%, 5%, and 7% wt%, while unmodified cement served as the control. Specimens were prepared and evaluated using SEM, EDS, and Raman spectroscopy for nanotube characterization and distribution, and tested for setting time, color, radiopacity, water sorption, and solubility according to standardized laboratory protocols.	Incorporation of TiO_2 nanotubes into the glass-ionomer cement was successfully confirmed and generally improved the material's physicochemical properties. TiO_2 addition reduced surface cracking, increased radiopacity, decreased solubility, and produced concentration-dependent changes in the cement structure. It slightly prolonged the initial setting time at higher concentrations and altered optical properties by reducing luminosity, while water sorption remained largely unaffected.	TiO_2 nanotube incorporation partially modified the glass-ionomer cement, improving some physicochemical properties and interacting with the cement matrix without affecting final setting time or water sorption.
Hepdeniz et al. (2021) [92]	To investigate whether adding TiO_2 nanoparticles to a conventional GIC and an amalgomer affects their composition and surface microhardness.	Two restorative materials (Ionofil U and Amalgomer CR) tested in control and TiO_2-modified forms. Disc-shaped specimens were prepared per group. Experimental groups had TiO_2 nanoparticles blended into the restorative powder	SEM/EDS confirmed uniform TiO_2 distribution and elevated titanium content in experimental groups. Microhardness slightly increased with adding TiO_2 in both materials, but remained statistically insignificant ($p>0.05$). Highest VHN:	Adding 3% TiO_2 nanoparticles modestly improved surface microhardness of both materials, though without statistical significance.

		at 3% w/w. All specimens assessed via SEM/EDS for surface morphology and elemental composition, and by Vickers hardness testing after 24-hour water storage.	Amalgomer experimental (84.34±4.33); lowest: Ionofil control (58.62±6.90). Between-material comparisons were statistically significant ($p<0.05$).	
Panahandeh et al. (2024) [93]	To evaluate how different concentrations of TiO_2 nanoparticles added to conventional GIC affect its flexural strength and surface hardness.	TiO_2 nanoparticles were blended into Fuji II GIC powder at 3, 5, and 10 wt%, and an unmodified control group. Material characterization by: XRD, FTIR, and SEM. Flexural strength was tested using three-point bending, and surface hardness via Vickers testing, both measured at 24 hours and one week.	SEM confirmed uniform TiO_2 distribution at 5 wt%. Flexural strength was significantly higher only in the 5% group vs. control and 3% groups ($p<0.05$); other comparisons non-significant. Surface hardness decreased with TiO_2 addition across all concentrations with 10% showing the lowest hardness values. Control group exhibited the highest hardness at 24 hours.	Only 5% TiO_2 improved flexural strength, while all concentrations reduced surface hardness, likely due to nanoparticle agglomeration.

Table S3. Quality Assessment.

Authors	Clearly stated aims/objectives	Detailed explanation of sample size calculation	Detailed explanation of sampling technique	Details of comparison group	Detailed explanation of methodology	Operator details	Randomization	Method of measurement of outcome	Outcome assessor details	Blinding	Statistical analysis	Presentation of results	SCORE
Assery et al. (2020) [61]	2	0	2	2	2	0	0	2	0	0	2	2	58,3
Cibim et al. (2017) [48]	2	0	2	2	2	0	2	2	0	0	2	2	66,67
Cvjeticanin et al. (2024) [62]	2	0	2	2	2	0	0	2	0	0	2	2	58,3
Fathi et al. (2022) [63]	2	0	2	2	2	0	0	2	0	0	2	2	58,3
Gjorgievska et al. (2020) [64]	2	0	2	2	2	0	0	2	0	0	2	2	58,3
Garcia-Contreras et al. [86] (2015)	2	0	2	2	2	0	0	2	0	0	2	2	58,3

Gjorgievska et al. [87] (2015)	2	0	2	2	2	0	0	2	0	0	2	2	58,3
Hamid et al. (2019) [65]	2	0	2	2	2	0	0	2	0	0	2	2	58,3
Hussein et al. (2022) [66]	2	0	2	2	2	0	0	2	0	0	2	2	58,3
Ibrahim et al. (2017) [67]	2	0	2	2	2	0	0	2	0	0	2	2	58,3
Ivanišević et al. (2021) [68]	2	0	2	2	2	0	0	2	0	0	2	2	58,3
Kantovitz et al. (2020) [69]	2	0	2	2	2	2	0	2	0	0	2	2	66,67
Laiteerapong et al. (2018) [70]	2	0	2	2	2	0	0	2	0	0	2	2	58,3
Mahendra et al. (2023) [71]	2	2	2	2	2	0	0	2	0	0	2	2	66,67
Mansoor et al. (2024) [72]	2	0	2	2	2	0	0	2	0	0	2	2	58,3

Mansoor et al. (2022) [73]	2	0	2	2	2	0	0	2	0	0	2	2	58,3
Meyer et al. (2025) [74]	2	0	2	2	2	0	0	2	0	0	2	2	58,3
Morales-Valenzuela et al. (2022) [75]	2	0	2	2	2	0	0	2	0	0	2	2	58,3
Ramić et al. (2024) [76]	2	0	2	2	2	0	0	2	0	0	2	2	58,3
Rangel-Coelho et al. (2024) [77]	2	2	2	2	2	0	0	2	0	0	2	2	66,67
de Gois Sena et al. (2024) [78]	2	0	2	2	2	0	2	2	0	0	2	2	66,67
Showkat et al. (2023) [79]	2	0	2	2	2	0	0	2	0	0	2	2	58,3
da Silva Morais et al. (2022) [80]	2	2	2	2	2	0	0	2	1	2	2	2	79,17

de Souza Araujo et al. (2021) [81]	2	2	2	2	2	0	2	2	0	0	2	2	75
Wassel et al. (2022) [82]	2	2	2	2	2	0	0	2	0	0	2	2	66,67
Karamüftüoğlu et al. (2026) [83]	2	1	2	2	2	0	0	2	1	0	2	2	66,67
Abozaid et al. (2026) [84]	2	0	2	2	2	0	0	2	0	0	2	2	58,3
Shubha et al. (2025) [85]	2	2	2	2	2	0	0	2	0	0	2	2	66,67
Ganesh et al. (2026) [88]	2	2	2	2	2	0	0	2	0	0	2	2	66,67
Garcia-Contares et al. (2014) [89]	2	0	2	2	2	0	0	2	0	0	2	2	58,3
Shahpaska et al. (2026) [90]	2	0	2	2	2	0	0	2	0	0	2	2	58,3

Kantovitz et al. (2023) [91]	2	2	2	2	2	0	1	2	2	2	2	2	87,5
Hepdeniz et al. (2021) [92]	2	0	2	2	2	0	0	2	0	0	2	2	58,3
Panahandeh et al. (2024) [93]	2	0	2	2	2	0	0	2	0	0	2	2	58,3
